# Ist2 promotes lipid transfer by Osh6 via its membrane tethering and lipid scramblase activities

**DOI:** 10.1126/sciadv.adz2217

**Published:** 2025-11-14

**Authors:** Alicia Fabbre, Camille Syska, Heitor Gobbi Sebinelli, Anna Cardinal, Maud Magdeleine, Noha Al-Qatabi, Juan Martín D’Ambrosio, Cédric Montigny, Guillaume Lenoir, Alenka Čopič, Guillaume Drin

**Affiliations:** ^1^ CNRS, INSERM, Institut de Pharmacologie Moléculaire et Cellulaire, Université Côte d’Azur, 660 route des lucioles, 06560 Valbonne, France.; ^2^ CNRS, Centre de Recherche en Biologie Cellulaire de Montpellier (CRBM), Université de Montpellier, Montpellier, France.; ^3^ CEA, CNRS, Institute for Integrative Biology of the Cell (I2BC), Université Paris-Saclay, Gif-sur-Yvette, France.

## Abstract

Lipid transfer proteins unevenly distribute lipids within the cell, which is crucial for its functioning. In yeast, Osh6 transfers phosphatidylserine (PS) from the endoplasmic reticulum (ER) to the plasma membrane (PM) by exchange with phosphatidylinositol 4-phosphate. We investigated why its activity depends on Ist2, an ER-resident lipid scramblase that connects the ER to the PM via an intrinsically disordered region (IDR). We found that Osh6, in a lipid-loaded state, binds the Ist2 IDR with micromolar affinity and functions at ER-PM contact sites only if its binding site within the IDR is sufficiently distant from the ER membrane. We determined, in reconstituted contact sites, that the association of Osh6 with the Ist2 IDR enables rapid, directed PS transfer. We identified the Ist2-binding site in Osh6 by molecular modeling and functional analyses. Last, we established that Ist2’s scramblase activity sustains Osh6-mediated PS transfer between membranes. Identifying these functional partnerships highlights why lipid transport processes are organized in membrane contact sites.

## Teaser

A TMEM16-like protein, via its lipid scramblase and membrane tethering functions, helps an ORP to convey lipids at contact sites.

## INTRODUCTION

Lipids are unevenly distributed within eukaryotic cells, which is essential for many cellular processes, like signal transduction or vesicular trafficking, as well as for cellular architecture (*1*–*3*). This is especially true for phosphatidylserine (PS): Like most other glycerophospholipids, PS is synthesized at the endoplasmic reticulum (ER), but its concentration in the ER membrane is low, whereas it is highly enriched (up to fivefold) at the plasma membrane (PM) and the endosomes (*4*). PS contributes to the negative charge of the PM and is critical for the recruitment and activation of various signaling proteins (*5*). How PS becomes selectively enriched at the PM was largely unknown until the finding that in yeast cells, two members of the oxysterol-binding protein (OSBP)–related protein (ORP) family (*6*), Osh6 and its close homolog Osh7, are lipid transfer proteins (LTPs) that carry PS from the ER to the PM (*7*). We have further shown that Osh6 exchanges PS for phosphatidylinositol 4-phosphate [PI(4)P], carrying PS from the ER to the PM, coupled with the downhill transport of PI(4)P from the PM to the ER (*8*). The PI(4)P concentration gradient is maintained thanks to the phosphatidylinositol 4-kinase Stt4, which generates PI(4)P at the PM in an adenosine 5′-triphosphate–dependent manner (*9*), and to the phosphatase Sac1, which degrades PI(4)P at the ER (*10*, *11*).

In parallel, it has been reported that human ORP5 and ORP8 function as PS/PI(4)P exchangers at ER-PM contact sites (*12*, *13*), where the distance between the ER and the PM is less than 20 nm (*14*). These proteins have a more elaborate organization than Osh6: In addition to a lipid transfer domain [i.e., OSBP-related domain (ORD)] closely related to that of Osh6, they contain a C-terminal transmembrane (TM) domain to anchor them to the ER and an N-terminal phosphoinositide-binding pleckstrin homology (PH) domain to target the PM. This molecular organization allows them to engage in ER-PM contact sites and efficiently transfer lipids. However, in yeast cells, Osh6 and Osh7 can likewise be observed at the cortical ER, representing sites of close apposition between the ER and the PM (*15*–*17*), although they comprise only a single ORD and have no tethering domains/motifs. We and others uncovered that the cortical localization of Osh6 depends on its interaction with Ist2 (*16*, *17*), one of the main proteins that scaffold ER-PM contact sites in yeast (*18*–*21*).

Ist2 consists of an N-terminal TM domain embedded in the ER membrane (590 amino acids), followed by an extended, disordered cytosolic region of ~360 amino acids (*22*). This intrinsically disordered region (IDR) permits Ist2 to interact with the surface of the PM via a short polybasic motif at the C terminus that can bind specifically to phosphatidylinositol 4,5-bisphosphate [PI(4,5)P_2_], a signaling phosphoinositide enriched in this membrane (*23*, *24*). Deletion of Ist2 leads to a decrease in the ER area associated with the PM, but most of these contact sites are still maintained by other ER-PM tethering proteins (*20*, *21*). By contrast, the function of the ER-embedded domain of Ist2 has long remained enigmatic. It comprises 10 predicted TM helices and bears homology to the transmembrane protein 16 (TMEM16) family, whose members have been shown to function as phospholipid scramblases, facilitating lipid flip-flop across the bilayer structure of the membrane (*25*, *26*). Although an initial study did not uncover any scrambling activity in proteoliposomes (PLs) prepared with Ist2 (*27*), two recent studies demonstrated that the ER domain of Ist2 has Ca^2+^-independent phospholipid scramblase activity that influences processes at the ER, like vesicular transport and lipid droplet biogenesis (*28*, *29*). Furthermore, cryo–electron microscopy analysis confirmed that the TM domain of Ist2 structurally resembles TMEM16 proteins (*29*).

Osh6 interacts with Ist2 by binding to a short motif in its disordered cytosolic tail and transfers PS at ER-PM contact sites (*16*, *17*). Mutations of Osh6 or Ist2 that prevent this interaction result in cytosolic localization of Osh6 and a substantial defect in the PS transfer to the PM (*16*). However, why Osh6 must form a complex with Ist2 to maintain efficient PS transfer remains enigmatic. Although membrane contact sites are considered essential hubs for interorganelle lipid transfer because many LTPs localize there (*30*), the advantage of this organization for the cell has been highly debated (*31*–*34*). Several possibilities have been proposed, which are not mutually exclusive.

First, it has been proposed that the localization of LTPs to membrane contact sites enables faster lipid transport. The idea is that a short distance between membranes might reduce the time needed for an LTP to move from one membrane to the other during a transport cycle; however, this hypothesis has not been addressed experimentally and remains disputed (*33*, *34*). A second hypothesis is that the confinement of LTPs to contact sites guarantees a high degree of accuracy in transport, as it would prevent mistaken delivery of their lipid ligands to other organelles (*31*, *32*). A third possible benefit is that the colocalization of LTPs to membrane contact sites enables or favors coordination between different lipid transport processes. In this respect, the interaction between Osh6 and Ist2 is particularly intriguing in light of the recently established lipid scrambling activity of Ist2. In eukaryotic cells, it is unclear whether PS is predominantly present in the cytosolic or luminal leaflet of the ER (*35*, *36*) and thus easily accessible to a cytosolic protein. Recent structural data on human PS synthase 1 indicate that PS is synthesized in the luminal ER leaflet (*37*), highlighting the need for PS scrambling at the ER. More generally, an important question is whether lipid scramblases and LTPs can function together to ensure robust lipid fluxes between cellular membranes while maintaining their bilayer architecture. For instance, cellular and structural data suggest that the rod-shaped LTP autophagy-related 2 (ATG2) protein works with two ER scramblases, TMEM41B and VMP1 (vacuole membrane protein 1), and a third scramblase, ATG9, embedded in the nascent autophagosome to promote autophagosome expansion with phospholipids made in the ER (*38*–*42*). Furthermore, Faesen’s group (*43*) recently demonstrated in vitro that ATG9, likely due to its scramblase activity, enhances the export of phospholipids by ATG2 from membranes in which it is present. These studies question whether scramblases are involved in other lipid transfer pathways or whether their role is specific to the formation of autophagosomal membranes.

Here, we have analyzed the functional coupling between Osh6 and Ist2 by combining biochemical approaches, in vitro reconstitution assays, cellular observations, and structural modeling. We demonstrate that Osh6 associates with the IDR of Ist2 with a micromolar affinity, independent of its loading with a lipid ligand. We test in cells how the Ist2 IDR contributes to PS transfer to the PM and show that PS transport is delayed if the Osh6-binding site is too close to the ER surface. Then, we recapitulate the capacity of Ist2 to connect ER- and PM-like membranes and to recruit Osh6 between these membranes. Next, we show that the association of Osh6 with the Ist2 IDR allows a rapid and directed flux of PS between membranes connected by the IDR. We further support this observation by generating Osh6 mutants that are unable to interact with Ist2 and do not show increased PS transfer activity between tethered liposomes. Last, by combining in vitro PS transfer assays with lipid scrambling assays using Ist2 PLs, we demonstrate that Ist2 sustains the PS transfer activity of Osh6 via its PS scrambling capacity. Collectively, these results provide the molecular basis for the functional coupling between an LTP and a dual membrane tether/lipid scramblase, which contributes to the proper distribution of PS in cells.

## RESULTS

### Analysis of the interaction between Osh6 and the disordered region of Ist2

**Fig. 1.**
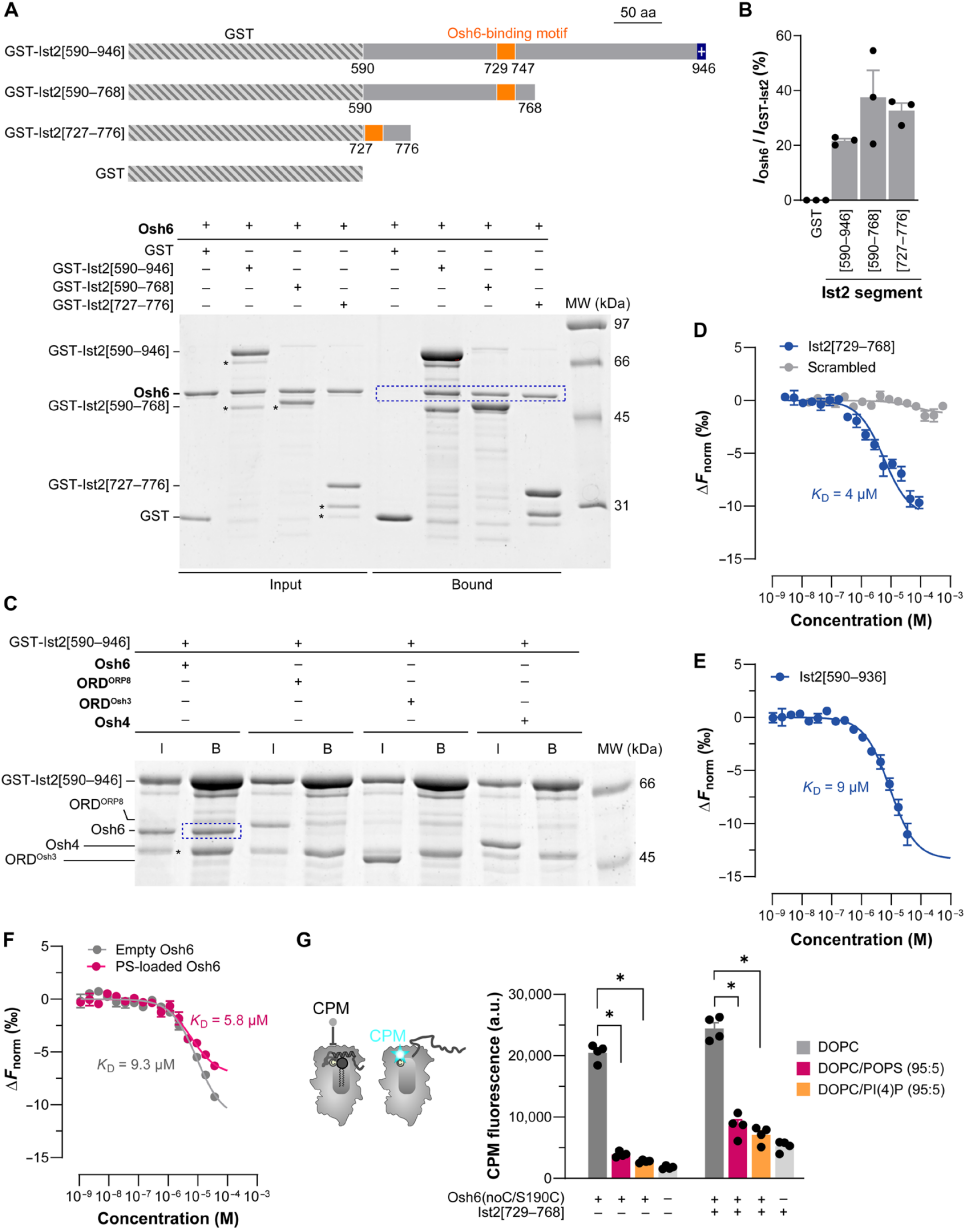
Interaction between Osh6 and the Ist2 IDR. (**A**) GST pull-down. Osh6 was mixed with [590–946], [590–768], or [727–776] segments of the Ist2 IDR appended to a GST, or GST alone, immobilized on glutathione beads. Input and bound fractions were analyzed by SDS-PAGE. Osh6 interacts with the three GST-tagged constructs (see inside the blue box) but not with GST. Stars, main contaminants in GST-tagged construct preparations. aa, amino acids; MW, molecular weight. (**B**) Quantification of GST pull-down. Data are shown as the means ± SEM (*n* = 3) with individual data points. (**C**) GST-Ist2[590–946], immobilized on beads, was incubated with Osh6, ORD^ORP8^, ORD^Osh3^, or Osh4. Input (I) and bound (B) fractions were analyzed using SDS-PAGE. (**D**) MST binding assay. Osh6 labeled with Alexa Fluor 647-C2 maleimide (AF647-Osh6, 20 nM) was mixed with different concentrations of Ist2[729–768] peptide or a scrambled version of this peptide at 25°C. An MST on-time of 5 s was set for the analysis, and baseline-corrected normalized fluorescence values (Δ*F*_norm_[‰]) were plotted against peptide concentration and fitted with a nonlinear regression model (*n* = 3 or 4). (**E**) MST binding assay with AF647-Osh6 and Ist2[590–936], performed as in (D) (*n* = 3). (**F**) Experiments performed as in (E) with AF647-Osh6, loaded or not with POPS (*n* = 3). Data are shown as the means ± SEM. (**G**) Accessibility assay. CPM (4 μM) was mixed with 400 nM Osh6(noC/S190C) incubated with DOPC liposomes, containing or not containing 5% POPS or PI(4)P, in the absence or presence of 40 μM Ist2[729–768] peptide. Control experiments were performed without Osh6(noC/S190C) and liposomes. Fluorescence at 465 nm (λ_ex_ = 387 nm) was measured 25 min after adding CPM (*n* = 3 or 4). a.u., arbitrary units. Data are shown as the means ± SEM with individual data points and were analyzed using an unpaired Mann-Whitney *U* test (*P < 0.05).

The C-terminal IDR of Ist2 contains about 360 amino acids. Evidence obtained in yeast cells and in vitro showed that Osh6 binds to a minimal motif in the region comprising residues 729 to 747 (*16*, *17*). However, no characterization exists of how Osh6 interacts with the full-length Ist2 IDR. To achieve this aim, we first used glutathione *S*-transferase (GST) pull-down assays. Three segments of the Ist2 IDR appended to an N-terminal GST were used as baits (Fig. 1A): a 727-to-776 segment encompassing the Osh6-binding motif of Ist2 as a positive control (*17*), a 590-to-768 segment in which this motif is at the C terminus of the construct and potentially easily accessible, and a 590-to-946 segment corresponding to the entire disordered region of Ist2. We found that all three Ist2 segments fused with GST recruited Osh6 to the beads with the same efficiency (Fig. 1, A and B). No binding was observed with GST alone or with an Ist2 construct with a truncated Osh6-binding motif (GST-Ist2[590–946]Δ727–749) (fig. S1). The same experiments with ORD^ORP8^, ORD^Osh3^, and Osh4 showed that these constructs were not recruited by the disordered region of Ist2, demonstrating the specificity of the Osh6:Ist2 interaction (Fig. 1C and fig. S2). These data show that Osh6 associates in vitro with the full-length Ist2 IDR by recognizing a binding motif site in its 727-to-776 segment.

Next, we measured Osh6’s affinity for the Ist2 IDR by microscale thermophoresis (MST). First, we produced a fluorescent version of Osh6 (AF647-Osh6) by coupling an Alexa Fluor 647-C2 maleimide dye to a unique cysteine (C262) lying at the protein surface (fig. S3, A to C; as shown later, we identified that the Ist2-binding site is away from this position; we were thus confident that the fluorescent dye would not perturb the capacity of Osh6 to interact with the Ist2 IDR). Then, we mixed AF647-Osh6 with an increasing concentration of a peptide corresponding to the Ist2[729–768] segment, encompassing the minimal Osh6-binding motif. MST traces indicated an interaction between Osh6 and this peptide, with a dissociation constant (*K*_D_) of ~4 μM (Fig. 1D and fig. S3D). A control experiment with a peptide corresponding to a scrambled version of the Ist2[729–768] segment revealed no binding, validating the assay’s specificity.

Next, we produced the Ist2 IDR devoid of a GST tag, with or without the C-terminal positively charged binding motif (Ist2[590–936] and Ist2[590–946]; fig. S3E) to perform MST measurements. However, we could only characterize Ist2[590–936], because Ist2[590–946] was not pure enough for spectroscopic analyses. We recorded the circular dichroism (CD) spectrum of Ist2[590–936], showing a minimum at λ = 198 nm (fig. S3F), which indicated that it was unfolded. Using dynamic light scattering (DLS), we found that Ist2[590–936], despite its smaller molecular weight compared to Osh6 (37 kDa versus 51.6 kDa), has a higher hydrodynamic radius (5.5 nm versus 3.8 nm) (fig. S3G), consistent with the notion that this Ist2 segment is an IDR. Last, we measured the affinity of AF647-Osh6 for Ist2[590–936] by MST and obtained a *K*_D_ of ~9 μM (Fig. 1E). These data indicated that Osh6 has no problem reaching its binding motif within the Ist2 IDR, with an affinity in the micromolar range.

Next, we examined how the Ist2 IDR binds to Osh6 in either an apo or lipid-bound state. To this end, following an established protocol (*44*), we prepared AF647-Osh6 loaded with 1-palmitoyl-2-oleoyl-*sn*-glycero-3 phospho-l-serine (POPS) or PI(4)P or in an empty form. We found that the affinity of AF647-Osh6 for Ist2[590–936] was similar (*K*_D_ ~ 6 μM versus 9 μM; Fig. 1F), whether it was in the apo form or bound to POPS. With the PI(4)P-loaded form of AF647-Osh6, we obtained variations in the MST signal that were too low to provide a *K*_D_ value. However, we found using GST pull-down assay that Osh6 loaded with PI(4)P can bind to the Ist2 IDR as efficiently as the empty or PS-bound form of Osh6 (fig. S3, H and I). Last, we assessed whether Osh6, associated with the Ist2 IDR, could bind to its lipid ligands. For this, we used an Osh6(noC/S190C) construct with a unique cysteine at position 190 that is solvent exposed only when the lipid-binding pocket of the protein is empty, as the molecular lid that gates this pocket is open in this case (*44*, *45*). This construct was added to liposomes doped or not with 5 mol % POPS or PI(4)P in the presence or absence of the Ist2[729–768] peptide. Then, 7-diethylamino-3-(4′-maleimidylphenyl)-4-methylcoumarin (CPM), a molecule that becomes fluorescent only when forming a covalent bond with accessible S190C, was added to each sample. After 25 min of incubation, we measured a fluorescence signal (*F*) with Osh6(noC/S190C) mixed with phosphatidylcholine liposomes, much higher than that measured in the control experiment without protein (Fig. 1G). In contrast, almost no increase in fluorescence was recorded when the protein was incubated with PS- or PI(4)P-containing liposomes. This indicated that Osh6(noC/S190C) remained mainly closed in the presence of its lipid ligands, as previously shown (*44*, *45*). Similar results were obtained when this protein was incubated with the Ist2[729–768] peptide. Together, these data established that the association of Osh6 with the Ist2 IDR and its loading with ligands are not mutually exclusive events and that Ist2 IDR binding to Osh6 occurs away from the Osh6 lipid-binding site.

### Influence of the Ist2 IDR on Osh6-mediated PS transfer in cells

**Fig. 2.**
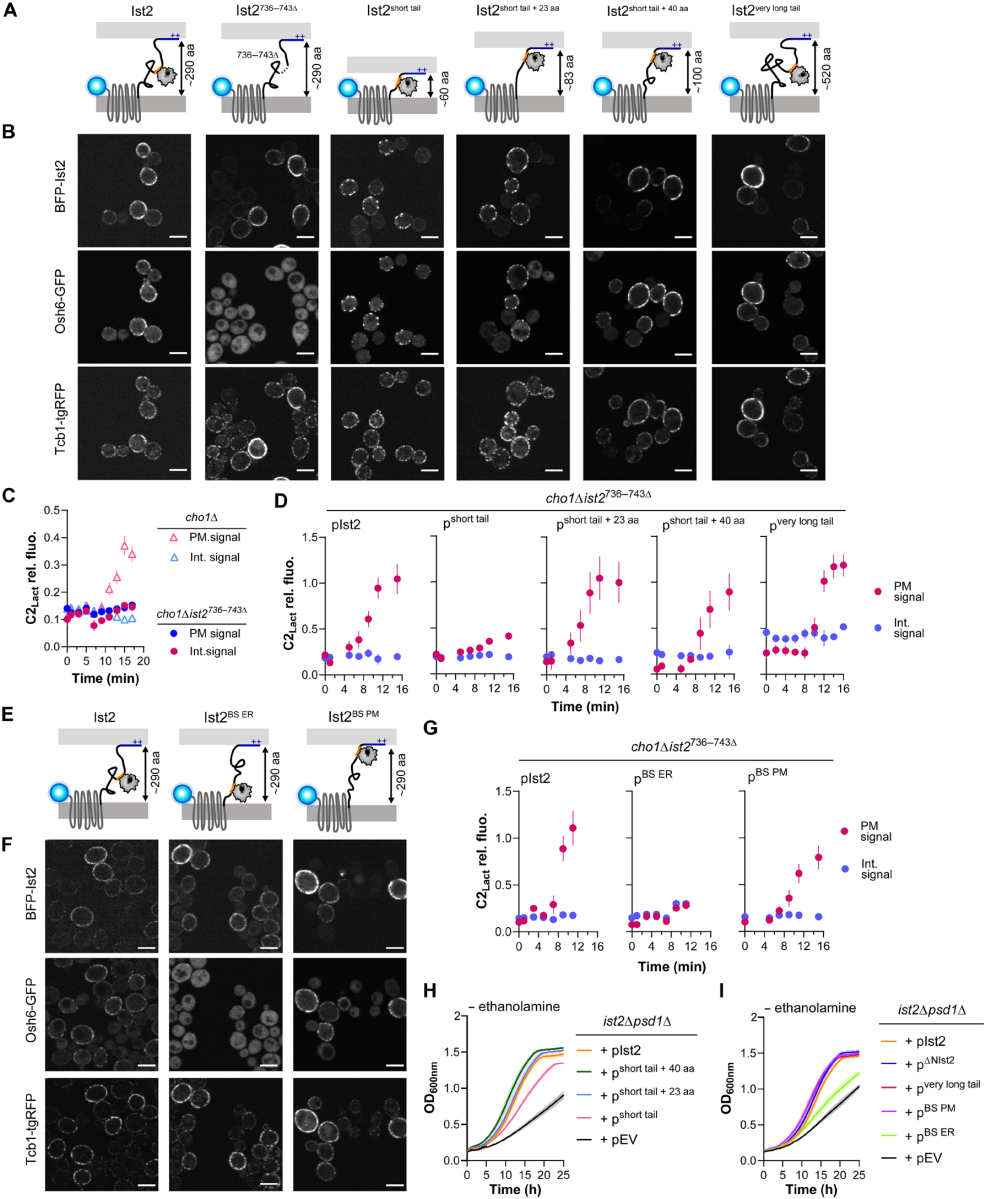
Evaluation of Ist2 IDR function in cells. (**A**) Diagrams of Ist2 WT and variants with truncated cytosolic tails, tagged N-terminally with BFP (blue sphere). The length of the tail between the ER-embedded TM region [1–590] and the extended PM-binding region ([878–946], dark blue) is indicated. Orange, Osh6-binding site. (**B**) Localization of BFP-Ist2 variants in *ist2*Δ cells and colocalization with Osh6-GFP and Tcb1-tgRFP. Scale bars, 5 μm. (**C**) PS transport assay. Localization of C2_Lact_-GFP was evaluated simultaneously in two strains lacking PS, *cho1*Δ, and *cho1*Δ*ist2*^*736–743*Δ^ every 2 min after adding lyso-PS (see also fig. S4D). The intensity of C2_Lact_-GFP signal is plotted over time, comparing internal peaks (blue symbols) and peripheral (PM) peaks (pink symbols), showing probe redistribution from internal (cytosolic and ER) toward peripheral (PM) peaks in *cho1*Δ (expressing Ist2-WT, triangles) but not in *cho1*Δ*ist2*^*736–743*Δ^ cells (round symbols). Data are represented as the means ± SEM (*n* = 10 to 15 cells) from one of two independent experiments. (**D**) Plasmids encoding BFP-Ist2 variants with truncated cytosolic tails were introduced into *cho1*Δ*ist2*^*736–743*Δ^*.* C2_Lact_-GFP localization was evaluated as in (C). Data are the means ± SEM (*n* = 10 cells) from one of two experiments. (**E** to **G**) Diagram of Ist2 variants with an Osh6-binding region [705–762] near the ER (after [1–589], Ist2^BS ER^) or near the PM (before [878–946], Ist2^BS PM^) (details in table S2). The localization of these variants (F) and PS transfer (G) was assessed as in (B) and (C), respectively. Scale bars, 5 μm. (**H** and **I**) Growth of *ist2*Δ *psd1*Δ cells with plasmids encoding BFP-Ist2 variants in minimal media without ethanolamine at 30°C [see control experiments with ethanolamine in fig. S4 (E to G)]. h, hours. Data are represented as the means ± SEM from triplicate cultures showing one of two independent experiments.

We next explored how the Ist2 IDR affects Osh6 localization and its PS transfer activity in a cellular context. For this, we constructed different variants of Ist2 with truncations of its IDR, which were tagged with blue fluorescent protein (BFP) at the N terminus (Fig. 2A). These variants were expressed in budding yeast from a low-copy plasmid and the native Ist2 promoter, as previously described (*16*). All variants contained an unperturbed C-terminal region, [878–946], to allow efficient binding to the PM (*24*, *46*). Deletion of this region resulted in improper localization of BFP-Ist2[1–877] in an *ist2*Δ background, likely implying protein aggregation (fig. S4A). Likewise, we could not express an N-terminal truncation mutant lacking the full TM domain of Ist2, BFP-Ist2[590–946]. In this case, the fluorescent signal was very low and again sometimes observed in punctate structures, suggesting protein aggregation (fig. S4B). These results show that the IDR of Ist2 must always be tethered to two membranes and that the lack of tethering leads to its aggregation in cells. In agreement, the localization of BFP-Ist2[1–877] can be rescued by the addition of a CAAX motif that leads to protein prenylation and attachment of the IDR to the PM via this lipid anchor (BFP-Ist2[1–877]PM) (fig. S4C). This result further underlines the importance of Ist2-IDR membrane attachment for its localization in cells, whereas the C-terminal region itself can be deleted.

When wild-type (WT) BFP-Ist2 was expressed in a strain with endogenously tagged Osh6-GFP (green fluorescent protein) and lacking the chromosomal copy of *IST2* (*ist2*Δ), it showed a patchy distribution at the cell periphery typical for ER-PM contact sites, consistent with the previously reported localization of Ist2 (*19*, *20*, *22*). Furthermore, it colocalized with another ER-PM contact site protein, tricalbin 1 (Tcb1), which was endogenously labeled with tagRFP (tgRFP). Osh6-GFP colocalized with Ist2 and Tcb1 and also showed some cytosolic signal (Fig. 2B, left panels). By contrast, when the binding site for Osh6 in the Ist2 IDR was truncated (Ist2^736–743Δ^) (*16*), Osh6-GFP was completely cytosolic, whereas the localization of Ist2 and Tcb1 was not affected. We then explored how deleting other regions of the Ist2 IDR, while preserving the full Osh6-binding region ([704–761]) (*16*), affects the localization of Ist2 and Osh6. It was previously shown that the cytosolic portion of the C-terminal tail of Ist2 could be substantially truncated without a noticeable effect on Ist2 localization until the truncation down to ~60 amino acids, where Ist2 showed a punctate localization (*16*, *22*). In agreement, our Ist2^short tail^ mutant, in which the cytosolic tail was reduced to a similar length (57 amino acids), containing just the Osh6-binding region [704–761], showed a more punctate localization at the cell periphery (Fig. 2B). Addition of 23 or 40 amino acids between the TM domain and the Osh6-binding region resulted in a normal Ist2 localization, and an increase in the length of the IDR by 230 amino acids (duplication of regions [590–704] and [763–877]) also did not substantially affect the Ist2 distribution nor its colocalization with Tcb1. In all cases, Osh6 colocalized with Ist2 at the cell periphery (Fig. 2B).

We next used a cellular PS transfer assay to assess the influence of the Ist2 IDR on the PS transfer activity of Osh6. This assay requires *cho1*Δ cells, which lack the sole PS synthase in yeast, Cho1, and therefore are devoid of PS (*47*). PS can be supplied exogenously in the form of 1-oleoyl-2-hydroxy-*sn*-glycero-3-phospho-l-serine (lyso-PS), which is transported to the ER and acylated into PS and whose transport back to the PM can be observed in real time using the fluorescent PS reporter C2_Lact_-GFP (*7*, *8*, *48*). As we have previously shown, C2_Lact_-GFP localizes to the PM within 15 to 30 min after lyso-PS addition in *cho1*Δ *IST2* cells but remains intracellular and localized to the ER in *cho1*Δ *ist2*^*736–743*Δ^ cells (*16*) (Fig. 2C and fig. S4D). We were not able to construct a *cho1*Δ *ist2*Δ strain because of synthetic lethality (*28*); therefore, the effect of BFP-Ist2 IDR truncation mutants on PS transport was assessed in the *cho1*Δ *ist2*^*736–743*Δ^ background (Fig. 2D). In this background, only Ist2^short tail^ showed a defect in PS transport, whereas the other truncation mutants as well as Ist2^very long tail^ supported PS transport to a similar extent to WT Ist2.

We also asked how the position of the Osh6-binding region within the Ist2 IDR affects Osh6 localization and its PS transfer activity. This binding region is positioned roughly in the middle of the IDR. We constructed two Ist2 mutants, Ist2^BS ER^, where the binding site was moved next to the TM region, and Ist2^BS PM^, where the binding site was moved just upstream of the C-terminal PM-binding region [878–946] (Fig. 2E). Both Osh6 localization and PS transport were affected by the Ist2^BS ER^ mutant but not by the Ist2^BS PM^ mutant (Fig. 2, F and G). The diminished recruitment of Osh6 by the Ist2^BS ER^ construct, which is not observed with the Ist2^short tail^ mutant, suggests that the surrounding environment of the binding region can also influence Osh6:Ist2 interaction.

An issue with our PS transport assay is that Ist2 IDR mutants were tested in the presence of Ist2^736–743Δ^, which cannot bind to Osh6 but could compensate for the IDR in trans, especially given that Ist2 dimerizes via its TM domain (*29*). We therefore used an independent genetic approach to evaluate how changes in the Ist2 IDR affect Osh6 activity and cellular PS homeostasis, assessing the genetic interaction of *ist2* mutants with the PS-decarboxylase mutant *psd1*Δ (*17*). As previously shown, the growth of *ist2*Δ *psd1*Δ cells is strongly inhibited in synthetic media lacking ethanolamine but is restored by the addition of ethanolamine (Fig. 2H and fig. S4E). Growth in this strain is rescued by the addition of WT Ist2 (pIst2) and by all Ist2 IDR mutants except for Ist2^short tail^ (Fig. 2H) and by Ist2^BS ER^ (Fig. 2I) or by ethanolamine (fig. S4, E to G), in agreement with the results of the PS transport assay. Together, this analysis shows that the majority of the Ist2 IDR is not required for Osh6 localization or PS transport activity in cells within the sensitivity of our assays, as long as the Osh6-binding region is sufficiently distant from the ER surface.

### Osh6 is recruited by the Ist2 IDR attached to ER or PM mimetic membranes

**Fig. 3.**
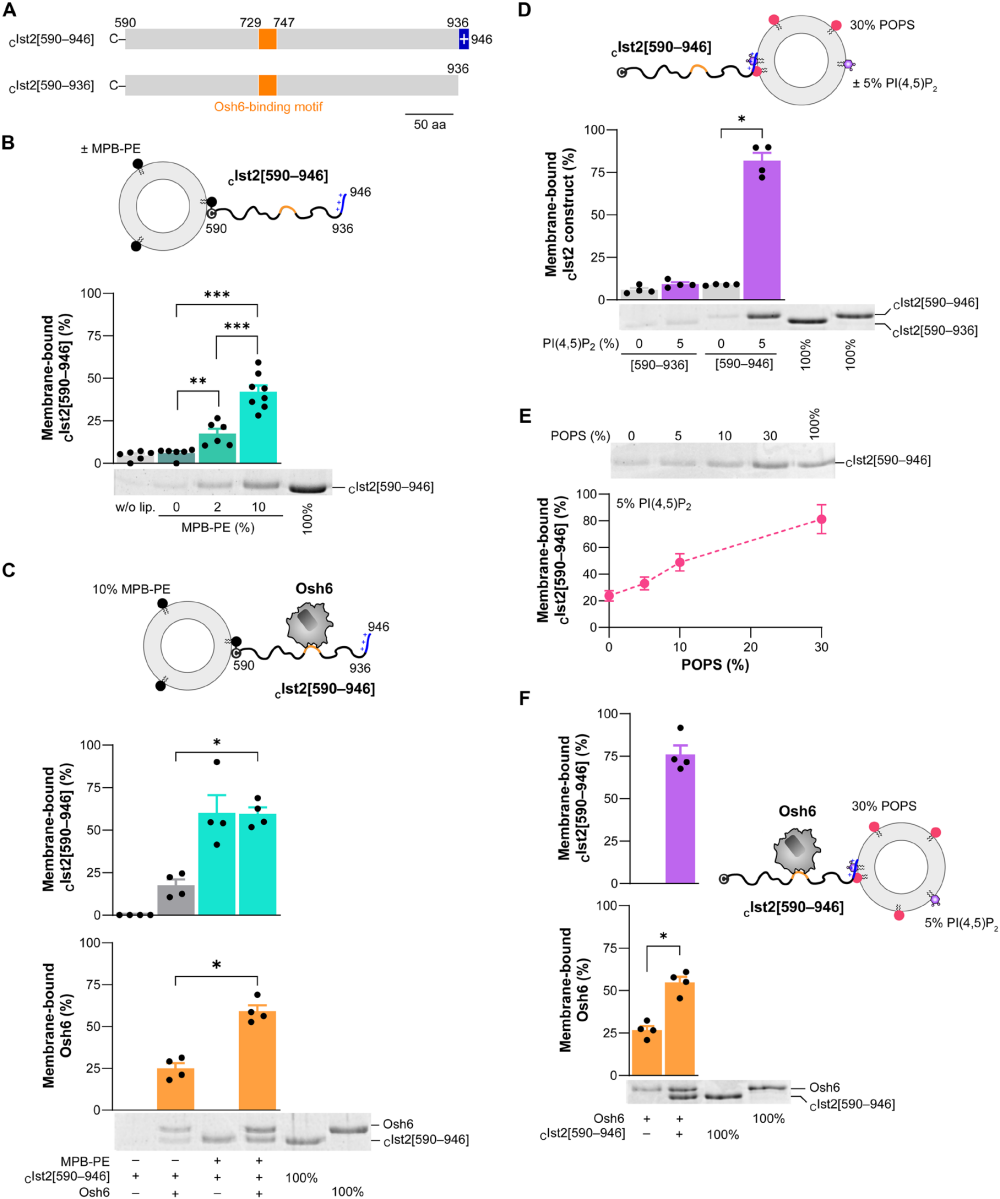
Osh6 is recruited by the Ist2 IDR, anchored by its N-terminal end to ER-mimetic liposomes or by its C-terminal end to PM-mimetic liposomes. (**A**) Flotation assays were performed with _C_Ist2[590–936] and _C_Ist2[590–946] constructs, corresponding to the Ist2 IDR, lacking or not lacking the C-terminal basic motif, with an extra N-terminal cysteine. (**B**) DTT-free _C_Ist2[590–946] (0.75 μM) was mixed with DOPC liposomes containing 0, 2, or 10% MPB-PE and 0.1% NBD-PE (750 μM total lipids) for 1 hour at 25°C. A control experiment was done without liposomes. After centrifugation, the liposomes were collected at the top of sucrose cushions and analyzed by SDS-PAGE. The amount of membrane-bound protein was determined using the content of lane 5 (100% total) as a reference (*n* = 6 to 8). (**C**) DTT-free _C_Ist2[590–946] (0.75 μM) was mixed with DOPC liposomes doped with NBD-PE, containing or not containing 10% MPB-PE. After 1 hour, DTT (2 mM) was added to stop the functionalization reaction, and the liposomes were mixed or not with Osh6 (0.75 μM) for 10 min. The amounts of membrane-bound _C_Ist2[590–946] and Osh6 were determined using the content of lanes 5 and 6 as references, respectively (*n* = 4). (**D**) _C_Ist2[590–946] or _C_Ist2[590–936] was mixed with POPC/POPS/NBD-PE (70:30:0.1) or POPC/POPS/PI(4,5)P_2_/NBD-PE (65:30:5:0.1) (*n* = 4). (**E**) _C_Ist2[590–946] was mixed with liposomes composed of POPC/PI(4,5)P_2_/NBD-PE (95:5:0.1) and increasing amounts of POPS (0, 5, 10, or 30%) at the expense of POPC (*n* = 4). (**F**) Osh6 (0.75 μM) was mixed in the presence or absence of an equivalent amount of _C_Ist2[590–946] with POPC/POPS/PI(4,5)P_2_/NBD-PE (65:30:5:0.1) liposomes (*n* = 4). Data are shown as the means ± SEM with individual data points and were analyzed using an unpaired Mann-Whitney *U* test (*P < 0.05, **P < 0.01,***P < 0.001).

To better understand why membrane tethering by the Ist2 IDR is necessary for PS transfer by Osh6, we decided to reconstitute this process in vitro. To do so, we first examined whether it was possible to anchor the N terminus of the disordered region of Ist2 to liposomes mimicking the ER membrane. We purified a _C_Ist2[590–946] construct (Fig. 3A and fig. S3E) with an extra N-terminal cysteine to covalently attach it to the surface of 1,2-dioleoyl-*sn*-glycero-3-phosphoethanolamine-*N*-[4-(*p*-maleimidophenyl)butyramide] (MPB-PE)–containing liposomes with an orientation comparable to that of the Ist2 IDR in a cellular context (it is worth noting that the Ist2 IDR does not contain endogenous cysteine). Next, we prepared liposomes composed of 1,2-dioleoyl-*sn*-glycero-3-phosphocholine (DOPC) to mimic the ER membrane, which is fluid and relatively neutral, and made of unsaturated lipids, additionally containing 0, 2, or 10% (mol/mol) MPB-PE. These liposomes (0.75 mM lipids) were mixed with dithiothreitol (DTT)–free _C_Ist2[590–946] (0.75 μM). Then, using flotation assays, we quantified how much protein was associated with the liposomes collected at the top of a sucrose gradient after ultracentrifugation. As expected, we found that _C_Ist2[590–946] was weakly anchored to liposomes consisting only of DOPC (1.4% of total protein, once the background signal measured without liposomes was subtracted) but ninefold more to liposomes containing 2% MPB-PE and 27-fold more to liposomes containing 10% MPB-PE (Fig. 3B). These results indicate that the _C_Ist2[590–946] construct can be covalently attached via its N-terminal end to liposomes containing 10% MPB-PE. Next, we tested whether the Ist2 IDR could recruit Osh6 to the vicinity of ER-like membranes. Liposomes, doped or not with 10% MPB-PE, were mixed with _C_Ist2[590–946], then treated with DTT to stop any subsequent coupling reaction with MPB-PE, and mixed with Osh6. Flotation assays showed that Osh6 was only slightly recruited to DOPC-only liposomes in the presence of _C_Ist2[590–946] but twofold more if this construct was attached to MPB-PE–containing liposomes (Fig. 3C). These observations suggest that the Ist2 IDR, anchored via its N terminus to the surface of neutral membranes, recruits Osh6.

In a second step, we examined whether the _C_Ist2[590–946] construct could interact with a membrane mimicking the inner leaflet of the PM via its C-terminal polybasic motif (*23*, *24*). To this aim, we analyzed by flotation assays to what extent _C_Ist2[590–946] and the construct lacking the polybasic motif, _C_Ist2[590–936] (fig. S3E), associated with liposomes composed of 70% 1-palmitoyl-2-oleoyl-*sn*-glycero-3-phosphocholine (POPC) and 30% POPS, doped or not with 5% PI(4,5)P_2_. We found that _C_Ist2[590–946] was firmly bound to liposomes containing PI(4,5)P_2_ but not to liposomes in which this lipid was lacking (Fig. 3D). In contrast, we found that _C_Ist2[590–936] did not associate with either type of liposome. Additional data showed that _C_Ist2[590–946] could substantially associate with liposomes containing only 5% PI(4,5)P_2_ but much more if these liposomes also included PS (Fig. 3E). We concluded that the Ist2 IDR efficiently binds to a membrane containing both PS and PI(4,5)P_2_ via its C-terminal polybasic motif, in support of previous results (*23*). Then, we mixed Osh6 with these PM-like liposomes in the absence or presence of _C_Ist2[590–946]. We observed that Osh6 associated significantly more with PM-like liposomes in the presence of the Ist2 IDR (Fig. 3F). Collectively, these data confirm that Ist2 can associate with a membrane mimicking the inner leaflet of the yeast PM via its C-terminal end and further demonstrate that it can recruit Osh6 in this configuration.

### Osh6 localizes at artificial ER-PM contact sites scaffolded by the Ist2 IDR

**Fig. 4.**
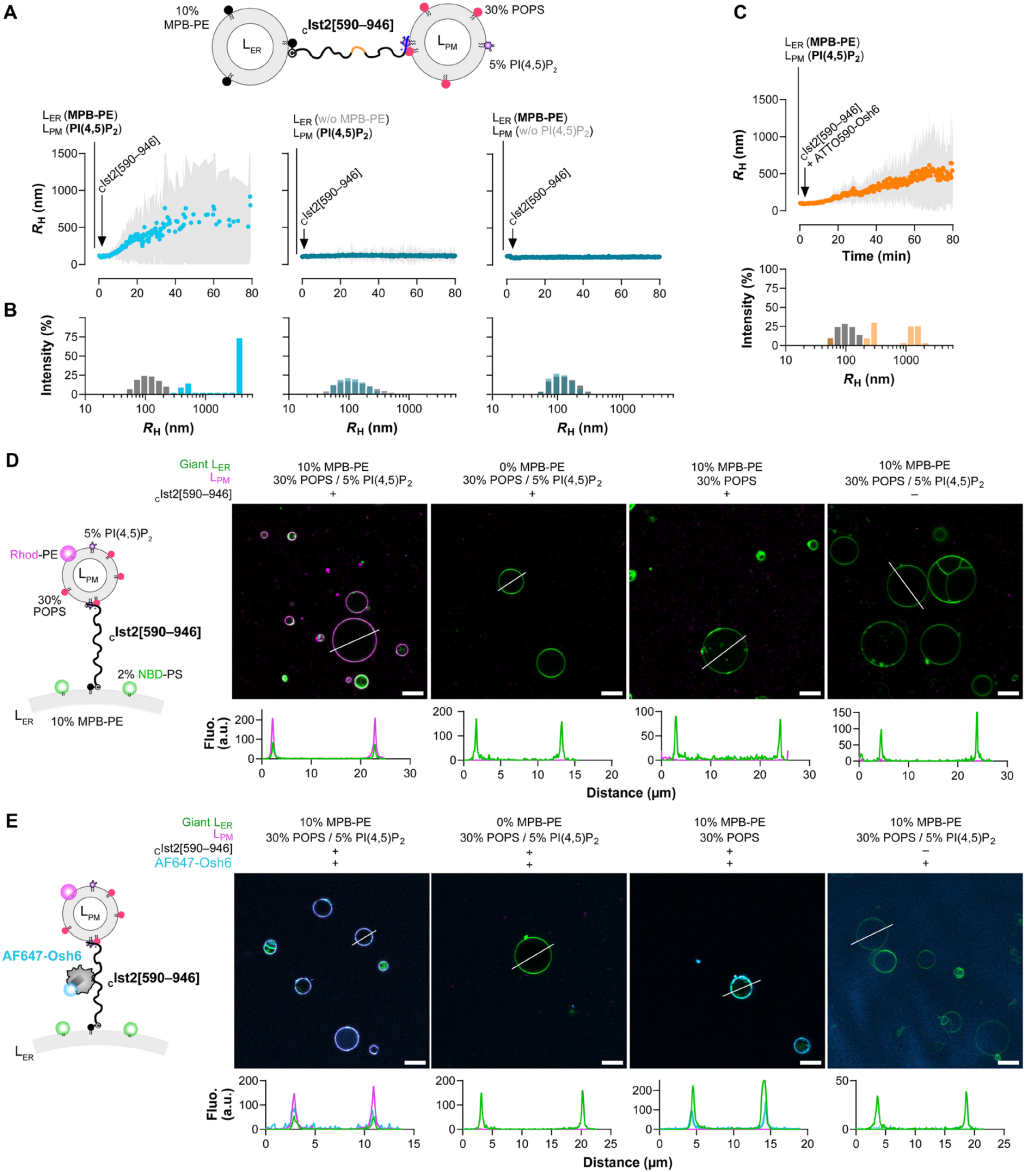
The Ist2 IDR can bridge ER- and PM-mimetic membranes and recruit Osh6. (**A**) DLS experiments. ER-like liposomes (L_ER_) composed of DOPC/MPB-PE/NBD-PE (90:10:0.1, 50 μM lipids) were mixed with an equivalent amount of PM-like liposomes (L_PM_) composed of POPC/POPS/PI(4,5)P_2_/NBD-PE (65:30:5:0.1) at 25°C. Then, DTT-free _C_Ist2[590–946] (0.5 μM) was mixed with liposomes. Control experiments were performed with L_ER_ liposomes devoid of MPB-PE or L_PM_ liposomes devoid of PI(4,5)P_2_. The mean radius (blue dots) and polydispersity (shaded area) of the liposome suspension were measured for 80 min. (**B**) Size distribution before (gray bars) and after the reaction (blue bars). (**C**) Aggregation kinetics measured with L_ER_ and L_PM_ liposomes mixed with _C_Ist2[590–946] and ATTO590-Osh6 (0.5 μM). The mean radius (orange dots) and polydispersity (shaded area) of the liposome suspension were measured for 80 min. (**D**) Confocal microscopy. Giant L_ER_ liposomes (~130 μM lipids) made of DOPC, doped with 2% NBD-PS, and containing or not containing 10% MPB-PE were incubated with small L_PM_ liposomes (L_PM_, 330 μM lipids) composed of 70% POPC, 30% POPS, and 0.25% Rhod-PE, doped or not with 5% PI(4,5)P_2_ (at the expense of POPC), in the absence or the presence of 1 μM DTT-free _C_Ist2[590–946]. All images were obtained using a Leica TCS SP8 (63×; NA, 1.4). The line scans show the fluorescence intensities of the green and magenta channels along the white line. Scale bars, 10 μm. (**E**) AF647-Osh6 (80 nM) was added to giant L_ER_ liposomes, doped with 2% NBD-PS, containing or not containing 10% MPB-PE, mixed with small liposomes composed of POPC/POPS/Rhod-PE (70:30:0.25), doped or not with 5% PI(4,5)P_2_, and DTT-free _C_Ist2[590–946]. A control experiment was carried out without _C_Ist2[590–946]. The line scans show the fluorescence intensities of the green, magenta, and blue channels along the white line*.* Scale bars, 10 μm.

Having established that _C_Ist2[590–946] can be attached to MPB-PE–containing liposomes via its N-terminal cysteine residue and binds to PS- and PI(4,5)P_2_-rich liposomes via its C-terminal basic motif, we next sought to determine whether it could connect these two types of liposomes. For this purpose, we measured by DLS whether _C_Ist2[590–946] could induce the aggregation of L_ER_ liposomes composed of DOPC and MPB-PE (90:10, 50 μM lipids) mixed with an equivalent amount of L_PM_ liposomes composed of POPC, POPS, and PI(4,5)P_2_ (65:30:5). Once mixed, in the absence of protein, the average hydrodynamic radius (*R*_H_) of these liposomes was ~100 nm with a low polydispersity (*P*_D_ < 50 nm). The addition of _C_Ist2[590–946] (500 nM) induced a substantial increase in the mean radius, reflecting its ability to promote liposome aggregation by connecting them (Fig. 4A). A more detailed analysis of the DLS measurements confirmed that at the end of the kinetics, massive liposome aggregates form, with a radius ranging from 400 to 4000 nm, at the expense of free liposomes (Fig. 4B). Control experiments with either L_ER_ liposomes devoid of MPB-PE or L_PM_ liposomes without PI(4,5)P_2_ showed, as expected, that no substantial aggregation occurred (Fig. 4, A and B). Next, we examined whether Osh6 affected liposome tethering by the Ist2 IDR. To facilitate this measurement, we used a construct (ATTO590-Osh6) devoid of free cysteine, meaning that no DTT had to be added to prevent the anchoring of Osh6 to the L_ER_ liposomes. When ATTO590-Osh6 was added to L_ER_ and L_PM_ liposomes along with _C_Ist2[590–946], we observed liposome aggregation to a level similar to that observed in our assays performed in the absence of Osh6 (Fig. 4C). We conclude that the Ist2 IDR can connect ER- and PM-like membranes irrespective of the presence or absence of Osh6.

To further validate our approach for reconstituting artificial ER-PM contacts with Osh6:Ist2 complexes, we visually examined whether Osh6 could be at the interface between ER- and PM-like membranes connected by the Ist2 IDR. We prepared giant L_ER_ liposomes composed of DOPC/MPB-PE/1-palmitoyl-2-(12-[(7-nitro-2-1,3-benzoxadiazol-4-yl)amino]dodecanoyl)-*sn*-glycero-3-phosphoserine (NBD-PS) (88:10:2, ~130 μM lipids) and incubated them with small L_PM_ liposomes (330 μM) composed of POPC/POPS/PI(4,5)P_2_/Rhodamine-PE [1,2-dioleoyl-*sn*-glycero-3-phosphoethanolamine-*N*-(lissamine rhodamine B sulfonyl); Rhod-PE] (65:30:5:0.25) in the presence of _C_Ist2[590–946] (1.5 μM). Observation by confocal microscopy revealed that the surface of the green giant liposomes was systematically coated with small red liposomes, as analyzed by line-scan analyses (Fig. 4D). The absence of either MPB-PE in the membrane of giant liposomes, PI(4,5)P_2_ in the membrane of small liposomes, or _C_Ist2[590–946] resulted in no recruitment of small liposomes to giant liposomes. Therefore, these data indicate that small liposomes with a PM-like composition can be recruited to the surface of giant liposomes coated with the Ist2 IDR. Upon addition of AF647-Osh6 to giant liposomes functionalized with _C_Ist2[590–946] and mixed with PI(4,5)P_2_-containing small liposomes, we observed far-red fluorescence overlapping with red and green fluorescence (Fig. 4E). The absence of MPB-PE in the membrane of giant liposomes or that of _C_Ist2[590–946] disrupted Osh6 recruitment. We concluded that Osh6 can localize between ER- and PM-like membranes connected by the Ist2 IDR.

### Ist2 IDR ensures a fast and directed Osh6-mediated PS transfer between connected membranes

**Fig. 5.**
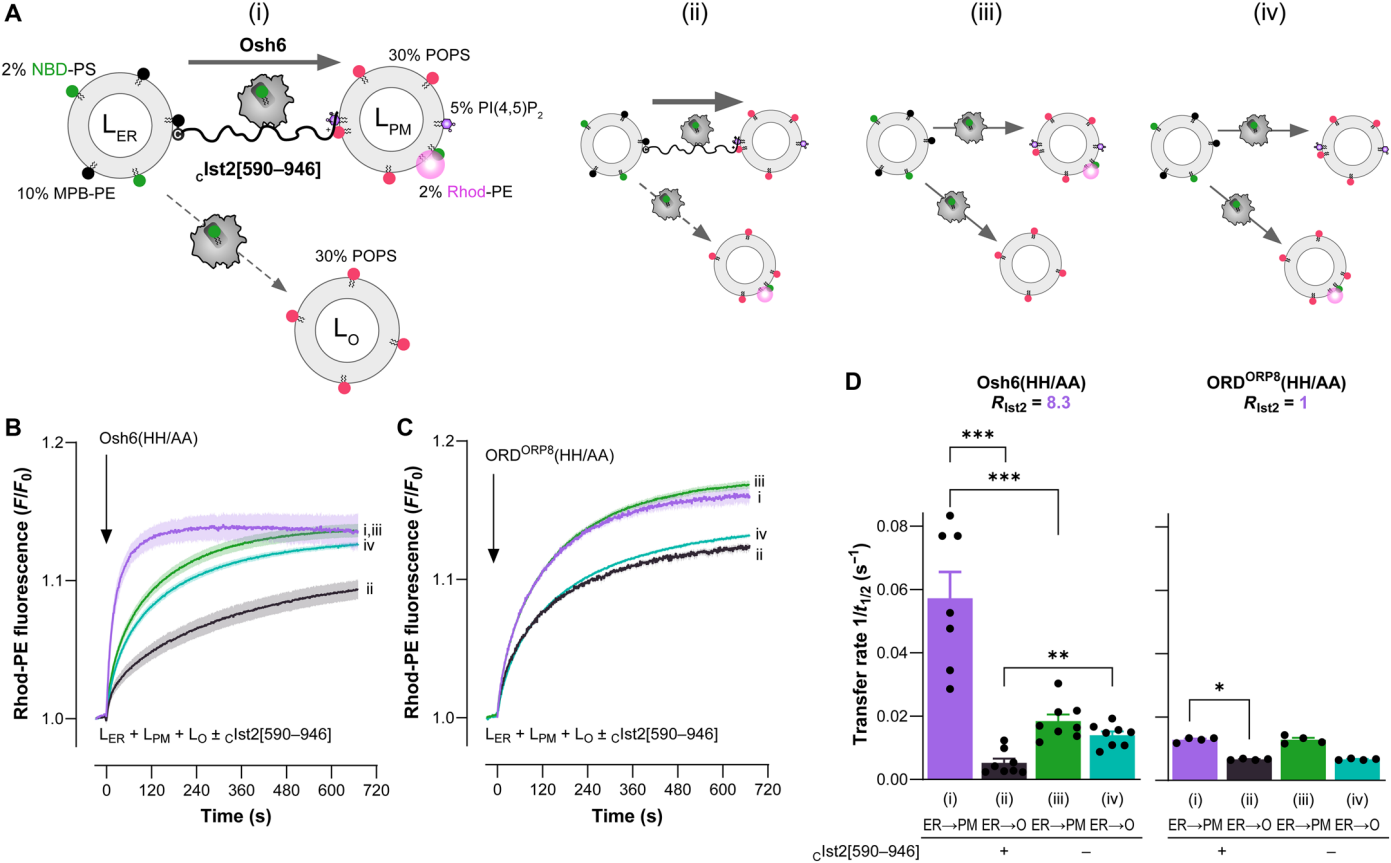
Tethering of membranes by the Ist2 IDR promotes fast and directed flux of PS by Osh6. (**A**) Graphical description of the NBD-PS transfer assays shown in (B) with three liposome populations under conditions (i) to (iv). (**B**) Under condition (i), Osh6(HH/AA) was added to L_ER_ liposomes (200 μM total lipids), composed of DOPC/MPB-PE/NBD-PS (88:10:2) and connected by _C_Ist2[590–946] (0.5 μM) to L_PM_ liposomes (200 μM), composed of POPC/POPS/PI(4,5)P_2_/Rhod-PE (63:30:5:2) in the presence of free L_O_ liposomes (200 μM) composed of POPC/POPS (70:30). The fast increase in rhodamine fluorescence (λ_ex_ = 460 nm, λ_em_ = 580 nm) over time corresponds to NBD-PS transfer from L_ER_ to L_PM_ liposomes. A mirror experiment (ii), in which Rhod-PE is incorporated in L_O_ liposomes and not L_PM_ liposomes, was conducted to measure the specific transfer of PS from L_ER_ to L_O_ liposomes. In the absence of _C_Ist2[590–946], Osh6(HH/AA) almost equally transfers PS to L_PM_ and L_O_ liposomes (iii and iv). Experiments were performed at 30°C. Each curve represents the means ± SEM of several kinetics (*n* = 7 to 8). (**C**) ORD^ORP8^(HH/AA) does not preferentially transfer PS between membranes connected by the Ist2 IDR, as there is no possible association between ORD^ORP8^ and the IDR (*n* = 4). (**D**) NBD-PS transfer rate (1/*t*_1/2_) measured with Osh6(HH/AA) and ORD^ORP8^(HH/AA) under conditions (i) to (iv). Data are shown as the means ± SEM with individual data points and were analyzed using an unpaired Mann-Whitney *U* test (*P < 0.05, **P < 0.01, ***P < 0.001). *R*_Ist2_ corresponds to the Ist2-dependent $\frac{\text{ER}\to \text{PM}}{\text{ER}\to O}$ transfer rate ratio.

The successful reconstitution of Osh6 recruitment to artificial ER-PM contact sites prompted us to investigate the ability of Osh6 to transfer PS in these contacts using a fluorescence assay based on NBD-PS. We and others have previously shown that Osh6 is not a PS/PI(4,5)P_2_ exchanger but that it can capture PI(4,5)P_2_ fortuitously in the absence of PI(4)P (*44*, *49*). Because the composition of our PM-like liposomes included PI(4,5)P_2_, we anticipated that the transfer of PI(4,5)P_2_ might interfere with the NBD-PS transfer activity of Osh6 and the Ist2 IDR’s ability to maintain contact sites. To mitigate this, we resorted to the Osh6(HH/AA) variant, less prone than the WT Osh6 to transfer PI(4,5)P_2_ (fig. S5A). GST pull-down and flotation assays showed that this construct binds well to the Ist2 IDR (fig. S5, B to D). To perform the transfer assay, we mixed L_ER_ liposomes containing MPB-PE and NBD-PS with L_PM_ liposomes enriched with POPS and doped with 5% PI(4,5)P_2_ and 2% Rhod-PE and L_O_ (O means other) liposomes only enriched with POPS. Next, the _C_Ist2[590–946] construct was added to the sample to connect the L_ER_ liposomes with the L_PM_ liposomes. Upon addition of Osh6(HH/AA), we measured a fast transfer of NBD-PS from L_ER_ to L_PM_ liposomes in the presence of L_O_ liposomes, as indicated by an increase in fluorescence resonance energy transfer (FRET) between NBD-PS and Rhod-PE (Fig. 5, A and B, trace i). The same experiment, but with Rhod-PE in L_O_ liposomes instead of L_PM_ liposomes, showed that NBD-PS transfer from L_ER_ to L_O_ liposomes was much slower than to L_PM_ liposomes (Fig. 5, A and B, trace ii). The prominent role of the Ist2 IDR in the Osh6-mediated lipid transfer was further substantiated by the fact that in its absence, NBD-PS was almost evenly transferred to L_PM_ and L_O_ liposomes (Fig. 5, A and B, traces iii and iv). By normalizing the FRET signal, we estimated that in the absence or presence of Ist2, approximately two-thirds of the NBD-PS pool initially present in the outer leaflet of L_ER_ liposomes, which is accessible to Osh6, was distributed between L_PM_ and L_O_ liposomes at the end of the kinetics, suggesting that our system reached near equilibrium (fig. S5E). By extracting ER → PM and ER → O transfer rates (1/*t*_1/2_) from the kinetics, we quantified that when L_ER_ liposomes were connected with L_PM_ liposomes by Ist2, the speed at which NBD-PS was transferred to L_PM_ and L_O_ liposomes increased and decreased, respectively, by approximately threefold. As a consequence, NBD-PS transfer was almost one order of magnitude faster toward L_PM_ liposomes than L_O_ liposomes (Ist2-dependent $\frac{\text{ER}\to \text{PM}}{\text{ER}\to O}$ transfer rate ratio *R*_Ist2_ = 8.3; Fig. 5D). When using Osh6 WT instead of Osh6(HH/AA), we measured lower NBD-PS transfer rates under all conditions, in line with the idea that PI(4,5)P_2_ might be trapped by Osh6 and interfere with its NBD-PS transfer activity, but we observed the same trend (fig. S5, E and F; *R*_Ist2_ = 7.6). On the contrary, with the ORD of ORP8 (bearing an HH/AA double mutation), we did not observe any acceleration or preference of transport toward L_PM_ liposomes connected to L_ER_ liposomes by _C_Ist2[590–946] (Fig. 5, C and D; *R*_Ist2_ = 1). These results suggest that the specific association of Osh6 with the disordered region of Ist2 enables rapid and accurate PS transfer between membranes connected by Ist2 without mistargeting to a third compartment.

### Osh6 mutations that prevent the interaction with the Ist2 IDR hinder Osh6-mediated PS transfer

**Fig. 6.**
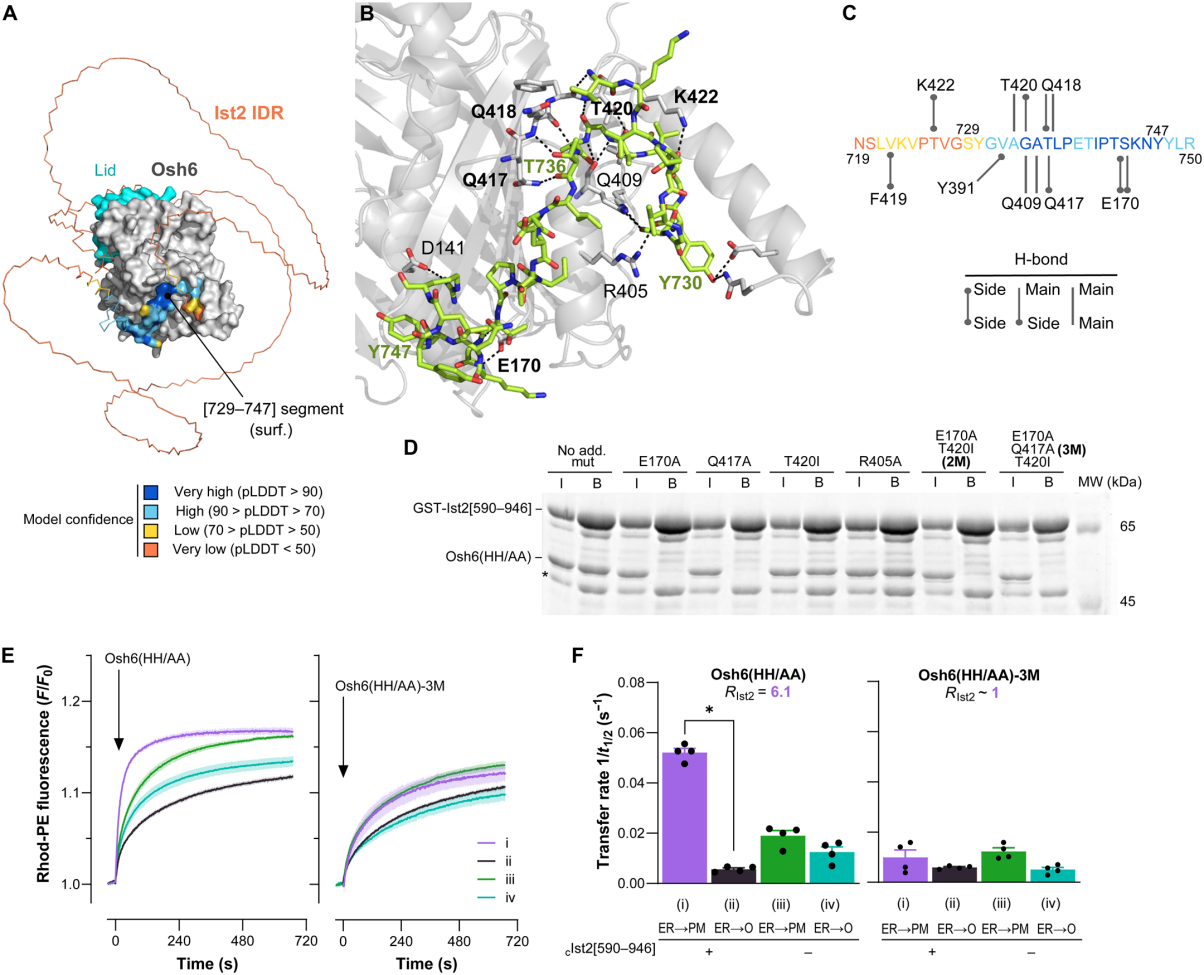
Identification of Osh6 mutants unable to preferentially transfer PS between Ist2-connected membranes. (**A**) AlphaFold3 model of the Osh6:Ist2 IDR complex. Osh6, without its N-terminal low-complexity region (35 amino acids, omitted for clarity), is represented as a gray surface. The lid, gating the lipid-binding pocket, is colored in cyan. The Ist2 IDR and Ist2[729–747] segment (minimal Osh6-binding motif) are represented in ribbon and surface mode, respectively, and colored according to the pLDDT per atom. (**B**) Close-up view of the Osh6:Ist2 IDR interface with Osh6 in cartoon mode and the Ist2[719–750] segment in stick mode (carbons in green, nitrogens in blue, and oxygens in red; reference residues are labeled in green). Dashed lines represent H-bonds between Osh6 and Ist2’s residues. The side and/or main chains of Osh6’s residues involved in these H-bonds are in stick mode (carbons in gray) and labeled in black (in bold, residues found in the five AlphaFold3 models to form H-bonds with the Ist2[719–750] segment). (**C**) H-bond network between the main and/or side chains of residues belonging to Osh6 and Ist2[719–750] segments (colored according to the pLDDT). Gray lines, H-bonds systematically identified in all models. (**D**) GST-Ist2[590–946] construct, on beads, was incubated with Osh6(HH/AA) bearing or not bearing additional mutations. Input (I) and bound (B) fractions were analyzed by SDS-PAGE. (**E**) NBD-PS transfer from L_ER_ liposomes to L_PM_ (i) or L_O_ liposomes (ii) measured with Osh6(HH/AA) or Osh6(HH/AA)-3M in the presence of _C_Ist2[590–946]. Similar measurements were done without _C_Ist2[590–946] (iii and iv). Each curve represents the means ± SEM of several kinetics (*n* = 4). (**F**) Transfer rate measured with each construct under conditions (i) to (iv). Means ± SEM with individual data points. Unpaired Mann-Whitney *U* test (*P < 0.05).

To further dissect the role of the Ist2 IDR in Osh6-mediated PS transfer, we designed Osh6 mutants that could not bind to it. To this end, we used AlphaFold3 (*50*) to build a model of the Osh6:Ist2 IDR complex using *Saccharomyces cerevisiae* Osh6 and Ist2[590–946] sequences. We obtained five top-ranked models (ranking score, 0.98 to 0.99) where the [719–750] segment of Ist2, encompassing the minimal Osh6-binding motif, was in close contact with Osh6’s surface (Fig. 6A and fig. S6A). Moreover, on the basis of the predicted aligned error (fig. S6B), the values predicted by the local distance difference test assigned to each Ist2 residue (Fig. 6A and fig. S6A) and the interface predicted template modeling score (0.79 for each model; fig. S6A), we concluded that the position and orientation of the [719–770] segment of the Ist2 IDR relative to Osh6, the folding of the Osh6-binding motif, and the Osh6:Ist2 binding interface were predicted with high confidence (*50*). An in-depth analysis of these models suggested that the [719–750] segment of Ist2 associates with an Osh6 surface region composed of residues belonging to the β3-β4 and β5-β6 loops and two helices, α8 and α9, which form the C-terminal end of the LTP. Notably, this region lies opposite the entrance of the lipid-binding pocket of Osh6. In all five models, we noted that two Osh6 residues (Q417 and Q418) are engaged in multiple H-bonds with the T736 residue in Ist2, which was previously found to be critical for the Osh6:Ist2 interaction (Fig. 6, B and C, and data S1) (*16*). Moreover, for all models, we observed that the backbone and/or side chains of other Osh6 residues (E170, Y391, Q409, T420, F419, and K422) are engaged in H-bonds with the same specific residues within the Ist2 IDR. In three models, the side chain of the D141 residue in Osh6, found to be necessary for Osh6:Ist2 interaction (*16*), is engaged in an H-bond with the side chain of the R750 residue in Ist2 (data S1). Thus, we obtained a model of Osh6:Ist2 IDR complex in good agreement with previous binding data (*16*, *17*) and identified Osh6 residues, in addition to D141 (*16*), that might be key for interacting with Ist2.

On the basis of AlphaFold3 predictions, we decided to mutate individually or in a combined manner Osh6 residues (E170, Q417, and T420) that, in all the models, form via their side-chain H-bonds with Ist2 residues (G734, T736, and S744) whose position, at the Osh6:Ist2 interface, was predicted with the highest confidence [predicted local distance difference test (pLDDT) value >90; Fig. 6C]. We also selected a residue (R405) whose side chain was predicted to be involved in an H-bond with Ist2 in only one model (data S1). Therefore, we purified Osh6(HH/AA) constructs with one additional mutation (E170A, Q417A, T420I, or R405A) or double (E170A/T420I called 2M) or triple mutations (E170A/Q417A/T420I, called 3M; fig. S6C). All these mutants were properly folded, as judged by their CD spectra (fig. S6D). In line with our predictions, we found that Osh6(HH/AA) constructs bearing an E170A or Q417A mutation, but also the Osh6(HH/AA)-2M and Osh6(HH/AA)-3M constructs, were unable to bind to the Ist2 IDR (Fig. 6D). Conversely, the mutation of T420 or R405 did not affect the association of Osh6 with the Ist2 IDR. To confirm these results at the cellular level, we analyzed the localization of Osh6(E170A) and Osh6-3M variants, chromosomally tagged with mCherry, and found that they were fully cytosolic. In contrast, Osh6 WT was mostly observed in patches at the cell cortex, indicative of its presence at ER-PM contact sites (fig. S7) (*15*–*17*, *45*). Control experiments showed that deletion of Ist2 rendered Osh6 WT cytosolic, as observed for the mutants when Ist2 is present. We conclude that residues E170 and Q417 are critical for Osh6 to recognize the Ist2 IDR. Last, we examined the ability of the 3M mutant to preferentially transfer NBD-PS from L_ER_ to L_PM_ liposomes connected by _C_Ist2[590–946] in the presence of free L_O_ liposomes. We found that contrary to Osh6(HH/AA), Osh6-3M did not preferentially transfer PS between liposomes connected by the Ist2 IDR; under all conditions tested (with or without _C_Ist2[590–946]), its transfer activity was similar to that of Osh6(HH/AA) measured between unconnected liposomes (Fig. 6, E and F; *R*_Ist2_ ~ 1). Together, these data not only validate our structural predictions of the Osh6:Ist2 interface but also confirm the specific requirement of the Osh6:Ist2 interaction for a fast and accurate, directed PS transfer between the ER and the PM.

### Osh6-mediated PS transfer can be coupled with the PS-scrambling activity of Ist2

**Fig. 7.**
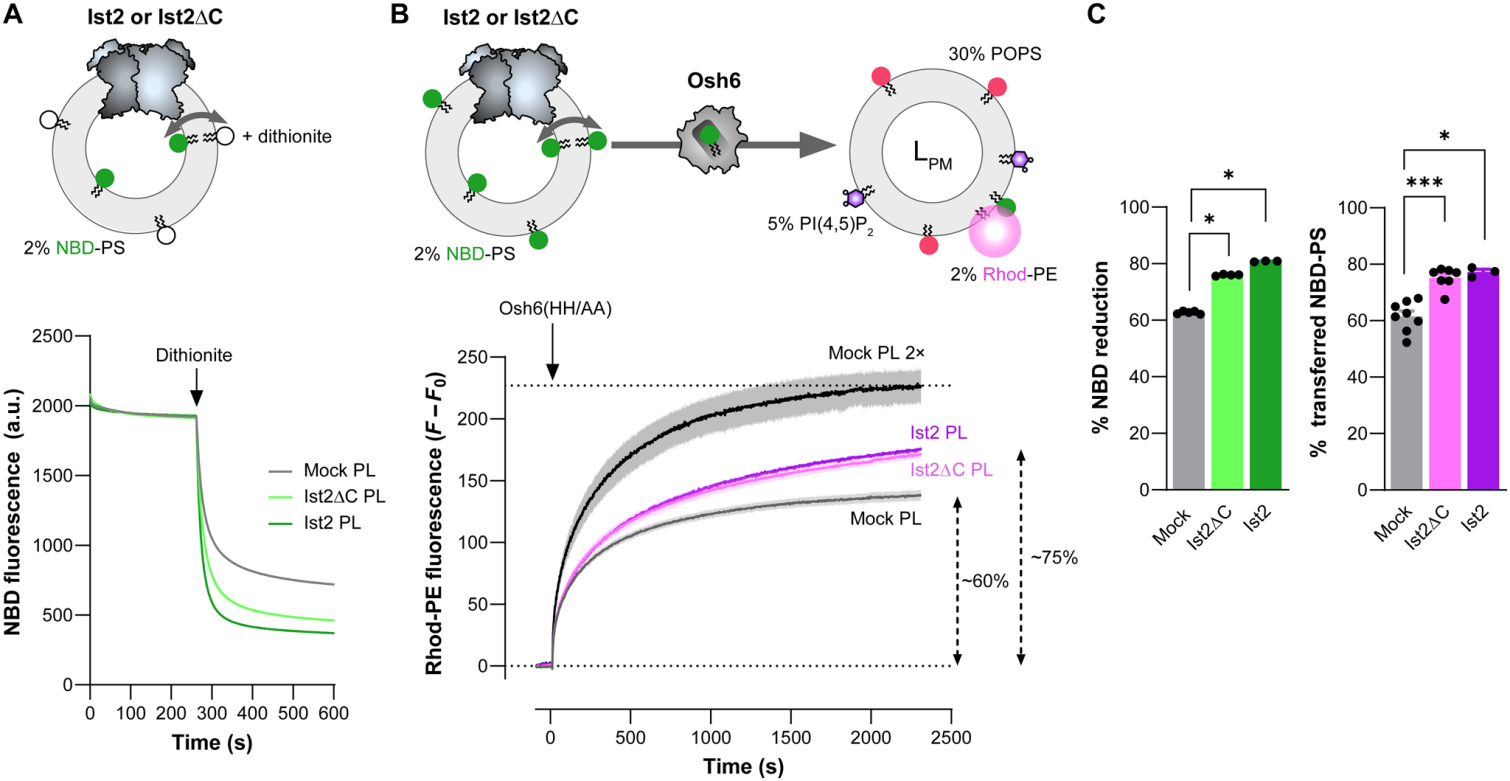
Coupling between the PS scrambling activity of Ist2 and the PS transfer activity of Osh6. (**A**) Scramblase assay. PLs composed of DOPC/NBD-PS (98:2), in which Ist2ΔC or Ist2 is reconstituted, or protein-free (“mock”) PLs have been diluted in HK buffer at 25°C under constant stirring. An equal amount of NBD-PS was present in the three conditions, as indicated by the NBD fluorescence level. After 5 min, dithionite (10 mM) was added, and the bleaching of NBD was measured over 5 min. Each curve represents the means ± SEM of several kinetics (*n* = 3 to 5). (**B**) Real-time NBD-PS transfer assays. NBD-PS–containing PLs, incorporating Ist2ΔC or Ist2 or no protein (“mock”), were mixed with L_PM_ liposomes (50 μM lipids) composed of POPC/POPS/PI(4,5)P_2_/Rhod-PE (63:30:5:2) in HK buffer at 25°C. An equal amount of NBD-PS is present under the three conditions. Osh6(HH/AA) was added, and NBD-PS transfer from PLs to L_PM_ liposomes was followed over time by measuring FRET (F) at 580 nm (λ_ex_ = 460 nm). Each curve corresponds to *F* − *F*_0_ values (*F*_0_, fluorescence measured before adding Osh6). For each condition, the final *F* − *F*_0_ was normalized to the final signal (*F*_max_ − *F*_0_) measured with twice the amount of protein-free PLs “mock PL 2×” to estimate the relative percentage of Osh6-mediated PS transfer. Each curve represents the means ± SEM of several kinetics (*n* = 3 to 8). (**C**) Percentage of NBD reduction by dithionite and relative percentage of Osh6-mediated PS transfer derived from measurements shown in (A) and (B) performed with the three types of PLs. Data are shown as the means ± SEM with individual data points and were analyzed using an unpaired Mann-Whitney *U* test (*P < 0.05, ***P < 0.001).

We have recently shown that Ist2 is a lipid scramblase at the ER (*28*). Therefore, a potential scenario is that Ist2, by equilibrating PS (among other phospholipids) between the leaflets of the ER membrane, fuels Osh6 for the transfer of PS to the PM. However, we did not observe any impact of the deletion of the Ist2 TM scrambling domain on the cellular PS levels or distribution (*28*). One issue may be the sensitivity/specificity of our assays. Given that deletion of the Ist2 TM domain is synthetically lethal with Cho1 deletion, we cannot implement our PS transfer assay in the ΔNIst2 strain. To explore whether Ist2 and Osh6 can partner for the redistribution of lipids between the ER and the PM, we measured the transfer of NBD-PS by Osh6 from PLs, prepared with the N-terminal part of Ist2, to liposomes mimicking the PM. First, we purified full-length Ist2 (hereafter called Ist2) and Ist2[1–600] (Ist2ΔC) fused to a biotin-acceptor domain after overexpression in *S. cerevisiae*. The two constructs were then reconstituted into PLs made of DOPC and doped with 2% NBD-PS, i.e., with a composition identical to the NBD-PS–containing donor liposomes we used so far in our transfer assays. For all conditions, most of the purified Ist2 was recovered after detergent removal with Bio-beads (fig. S8A). DLS indicated that the average *R*_H_ values were ~62, 56, and 64 nm for Ist2, Ist2ΔC, and protein-free (“mock”) PLs, respectively (fig. S8B). Then, we conducted scramblase assays in which dithionite was used to selectively bleach the fluorescence of NBD-labeled lipid present in the outer leaflet of the PLs (*51*). Adding dithionite to “mock” PLs provoked a ~63% reduction in the NBD signal, indicative of the percentage of NBD-PS accessible to the reducing agent. With Ist2 and Ist2ΔC PLs, the fluorescence was reduced by 81 and 76%, respectively, indicating that more NBD-PS was accessible on the outer leaflet (Fig. 7, A and C). These values, although lower than those obtained when Ist2 and Ist2ΔC were reconstituted in POPC/POPS PLs (fig. S8, A and C), as we have recently shown (*28*), indicated that Ist2 and Ist2ΔC exhibit robust scramblase activity in ER-like membranes.

Having established the scramblase activity of Ist2, we measured the PS transfer assay of Osh6 added to “mock,” Ist2ΔC, or Ist2 PLs (~50 μM lipids) preincubated with a similar amount of L_PM_ liposomes composed of POPC/POPS/PI(4,5)P_2_/Rhod-PE (63:30:5:2). The amount of NBD-PS was the same under all conditions (fig. S8D). With “mock” PLs, we measured a rapid increase in FRET signal from NBD-PS to Rhod-PE, reflecting the transfer of NBD-PS from PLs to L_PM_ liposomes, which reached near equilibration after 1 hour (Fig. 7B). When Osh6 was added to Ist2ΔC or Ist2 PLs, we measured a higher increase in FRET signal within the first minutes; moreover, this signal kept increasing even after 1 hour without reaching a plateau. These kinetics data suggested that the scramblase activity of Ist2 and Ist2ΔC sustained NBD-PS transfer by Osh6 to L_PM_ liposomes.

To strengthen these results, we first assessed by a collisional quenching assay that ~50% of the NBD-PS in the membrane of “mock,” Ist2ΔC, or Ist2 PLs was protected from iodide, indicating that this lipid was distributed equally between the membrane leaflets of PLs for each preparation (fig. S8E). Second, we verified that Osh6 did not affect the scramblase activity of Ist2 (fig. S8F). Third, we mimicked a situation where all the PS molecules would already be in the outer leaflet of the PLs before adding Osh6. To do so, we measured PS transfer using twice the amount of “mock” PLs and observed a transfer of PS higher than that observed with Ist2 and Ist2ΔC PLs within a few seconds (“mock PL × 2”; Fig. 7B). This meant that the effect seen with Ist2 and Ist2ΔC PLs was not due to a preexisting availability of PS that would be higher with these PLs. Instead, we concluded that it was due to the scramblase’s capacity to replenish the PS pool in the outer leaflet of PLs while Osh6 was functioning. The amount of NBD-PS transferred by Osh6 to L_PM_ liposomes remained lower than that measured under the “mock PL × 2” condition, likely reflecting the fact that the scrambling of NBD-PS in Ist2/Ist2ΔC PLs was incomplete (Fig. 7A). Last, by normalizing the PS transfer data, we found that Osh6 transferred up to ~15% more PS from Ist2/Ist2ΔC PLs than from “mock” PL, which was in line with the dithionite assay showing that up to ~20% more NBD-PS was accessible because of the Ist2 scrambling activity (Fig. 7C). By repeating the scramblase and NBD-PS transfer assays with PLs prepared with Ist2 at different protein-to-lipid ratios (0, 7, and 28 mg mmol^−1^ protein/lipid ratios), we found that if more NBD-PS was accessible at the surface of PLs because of Ist2’s activity, more NBD-PS was transferred by Osh6 to L_PM_ liposomes (fig. S8G). From these experiments, we conclude that Ist2 can augment the PS pool accessible to Osh6 in ER-like membranes and suggest that the PS transfer activity of Osh6 may be coupled to the Ist2 scramblase activity.

## DISCUSSION

**Fig. 8.**
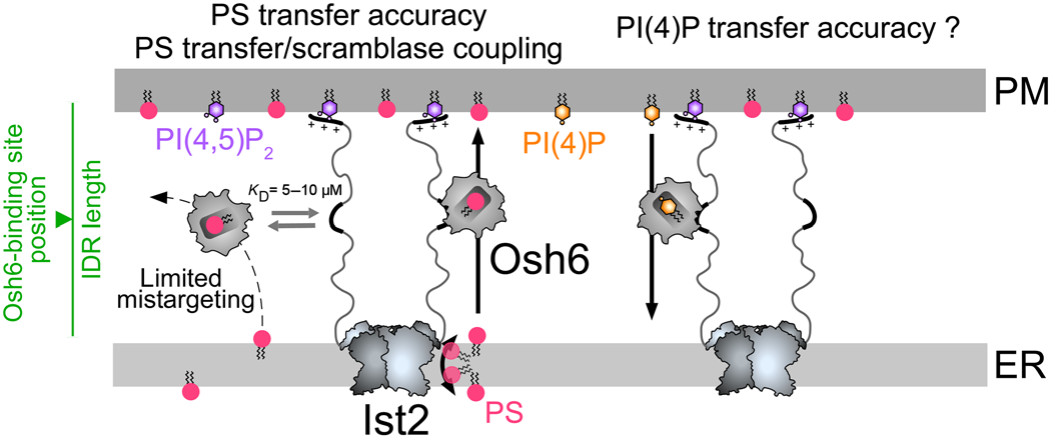
Model of the functional partnership between Osh6 and Ist2. Osh6 can bind to the Ist2 IDR in a PS-bound state and therefore transfer PS from the ER to the PM in a fast and directed manner, with a limited capacity to deliver PS by mistake into another cellular membrane. Moreover, the PS transfer activity of Osh6 can be coupled with the PS scrambling activity of Ist2. The length of the Ist2 IDR and the position of the Osh6-binding site within it are critical factors that affect the efficiency of Osh6-mediated PS transfer. Osh6 in a PI(4)P-bound state can also bind to the Ist2 IDR. Whether this association allows for a directed PI(4)P by Osh6 from the PM to the ER needs further investigation.

An important and emerging issue in cell biology is to understand why LTPs are localized to contact sites and how their concerted action with other lipid transporters and membrane tethering factors affects intracellular lipid distribution and metabolism. Here, we dissect in vitro how Osh6 associates with the IDR of Ist2. Using cell-based assays, we also report that the length of the Ist2 IDR and the position of the Osh6-binding site are essential for PS transport at ER-PM contact sites. We recapitulate PS transfer from the ER to the PM using purified components and determine that the association of Osh6 with Ist2 markedly increases the efficiency of PS transfer between membranes connected by the Ist2 IDR. Moreover, we demonstrate that the PS transfer activity of Osh6 and the scramblase activity of Ist2 can be coupled. These results allow us to propose a model on the functional partnership between the two proteins (Fig. 8).

First, we quantified how Osh6 associates with a purified form of the full-length Ist2 IDR, which we confirmed to have no secondary structure and a hydrodynamic radius close to that predicted or obtained for an IDR of similar length in solution (e.g., the 351-amino-acid-long IDR of GHR-ICD with an *R*_H_ = 5.08 nm) (*52*, *53*). The affinities of Osh6 for the full-length IDR or a peptide encompassing the Osh6-binding site were similar (*K*_D_ = 4 to 9 μM) and close to those measured by Dutzler and co-workers (*29*) between Osh6 and short segments of Ist2 IDR using calorimetry (*K*_D_ = 0.71 to 1.1 μM). This suggests that Osh6 has no difficulty reaching its binding site at the center of the Ist2 IDR, i.e., in the core of a region that likely forms a molten globule, as suggested by our structural models. Moreover, regardless of whether it is empty or loaded with a lipid ligand, Osh6 interacts with the Ist2 IDR in a similar manner, and this interaction does not affect how Osh6 traps lipid ligands. This means that Osh6 can operate lipid exchange cycles while interacting with Ist2, which maintains its localization at contact sites.

What can the measured affinity of Osh6 for Ist2 tell us about the association of the two proteins in the yeast cell? The median volume of a budding yeast cell is 44 fl, corresponding to a sphere of 4.4 μm in diameter (*54*). For a yeast of this size, the PM area is estimated to measure 102 μm^2^ (*55*). Considering that 4100 Ist2 copies per cell (www.yeastgenome.org/) and all the Ist2 proteins are at ER-PM contact sites, i.e., between two membranes that are 20 nm apart (*19*), the local Ist2 concentration there could be about 3.4 μM. In contrast, the cytosolic concentration of Osh6 (3600 copies per cell) is 135 nM. Considering a 1:1 binding model and a *K*_D_ between 1 and 10 μM, the fraction of Ist2-bound Osh6 in ER-PM contact sites could be 25 to 75% at the steady state. This fits well with cellular observations that Osh6 is partly cytosolic and partly localized to the cortical ER (*15*–*17*, *45*).

In yeast, Ist2 constructs with a shorter IDR can still connect the ER and the PM if the IDR is long enough to reach across the ER-PM contact sites, in agreement with previous work (*22*). These observations are also consistent with analyses of how the length of IDRs with membrane-binding modules influences their tethering activities (*56*). Intriguingly, changing the length of the Ist2 IDR can strongly affect the PS transfer activity of Osh6, even if its interaction with Ist2 is reversible. Notably, Osh6 functions optimally in our assays when it can interact with an 83-amino-acid or longer version of the Ist2 IDR but not with a 60-amino-acid version, although the latter can still connect the ER to the PM and recruit Osh6. We can deduce that it is sufficiently long [22 nm, considering 0.38 nm per residue for a fully extended configuration (*57*)] to span the ER-PM gap [ranging from 16 to 34 nm, with an average of 23 ± 5 nm (*18*)]. This same simple calculation suggests that Osh6, once bound to this short IDR, would be trapped at a maximum distance of ~10 nm from the ER surface and thus would not be able to reach the PM, explaining its loss of activity. This is reminiscent of the analysis by Gatta and co-workers (*58*) of the yeast LTP Ysp2, which showed that Ysp2 cannot transfer sterol at ER-PM contacts if its transfer module is linked to the ER by an IDR of less than 40 amino acids (15 nm).

By contrast, relocating the Osh6-binding motif just upstream of the C-terminal PM-binding segment of the Ist2 IDR has only a weak impact on Osh6 activity. This difference may be due to the reversible binding of the C-terminal Ist2 region to the PM, preventing the trapping of Osh6 at the PM. Notably, we could not test the effect of detaching the Ist2 IDR from the ER or the PM on Osh6 function because the corresponding truncated Ist2 constructs were mislocalized in cells and appeared to aggregate, highlighting the importance of carefully assessing protein localization in functional assays. For the same reason, it is currently difficult to interpret our results with the Ist2 mutant in which the Osh6-binding site was moved close to the ER, which localized correctly but only poorly recruited Osh6. However, this result indicates that the environment surrounding the Osh6-binding site also influences the Osh6:Ist2 interaction, as previously observed for the pairing of VAP (vesicle-associated membrane protein–associated protein) with various LTPs (*59*, *60*).

Jointly, these results suggest that the association of Osh6 with Ist2 at ER-PM contact provides a functional advantage, provided that Osh6 can reach both membranes. Moreover, they suggest that the association time between Osh6 and the Ist2 tail exceeds the time necessary for an intermembrane lipid transfer event. We conclude that thanks to its affinity for Ist2, Osh6 might function as an LTP capable of simultaneously transferring lipid and tethering membranes (e.g., ORP5/8) at contact sites.

We next reconstituted ER-PM contact sites in vitro by anchoring the Ist2 IDR via its N terminus to neutrally charged liposomes with low lipid packing, mimicking the ER membrane, and to liposomes with higher lipid packing, rich in PS with a minute amount of PI(4,5)P_2_, mimicking the PM. In line with previous reports (*23*, *24*), we observed that the membrane-binding capacity of the C-terminal motif of the Ist2 IDR strongly depends on PI(4,5)P_2_, which explains why Ist2 specifically anchors the ER to the PM. Next, we established that Osh6 could be recruited to these artificial contact sites in an Ist2 IDR–dependent manner. In the absence of the Ist2 IDR, Osh6 binds weakly to ER- and PM-like membranes. This suggests that even when associated with Ist2, Osh6 would maintain its ability to associate transiently with these membranes, which is necessary for its transfer activity (*45*).

Having reconstituted the Osh6:Ist2 complex at contact sites and knowing the selectivity of the Ist2 IDR for PI(4,5)P_2_, we could then measure Osh6-mediated PS fluxes between ER- and PM-like liposomes [containing PS and PI(4,5)P_2_] connected by Ist2 in the presence of a third population of liposomes, devoid of PI(4,5)P_2_. In this experimental setup that recapitulates the multicompartmentalized organization of a cell, we found that the specific association of Osh6 with the Ist2 IDR allows for a fast transfer of PS between connected membranes while preventing Osh6 from transferring PS toward another compartment. In the absence of interaction, PS was slowly transferred to all membranes. This matches well with our observation that the lack of Ist2 or Osh6:Ist2 association leads to a defect in the delivery of PS by Osh6 to the PM (*16*); likely, Osh6 slowly spreads PS toward different organelles and membranes. Furthermore, published data suggest that Osh6 transfers PI(4)P to the ER from the PM but not from the Golgi, although PI(4)P is also prominent in this organelle (*8*, *10*, *61*, *62*). Given that Osh6 bound to PI(4)P can associate with the Ist2 IDR, Ist2 might also ensure a directed PI(4)P transfer at ER-PM contact sites, locally coupled with PS transfer, and therefore efficient PS/PI(4)P exchange (Fig. 8). Further work is needed to confirm this. That said, our data demonstrate that the localization of LTPs at contact sites enables rapid and accurate lipid transfer between two membranes within the complex cellular environment.

So far, in vitro studies focused on LTPs (OSBP, STARD3, E-Syt1, and GRAMD1b) with a dual ability to tether membranes and carry lipids, showing that these LTPs efficiently transfer lipids between liposomes once they connect them (*63*–*66*). In other studies, lipid-transfer modules anchored to donor liposomes delivered lipids efficiently to acceptor liposomes only when both liposomes were brought in close proximity by a tethering factor (*67*–*69*). Consequently, it was impossible to distinguish whether lipid transfer was enhanced because of the concentration of LTPs between membranes, because of a shorter intermembrane distance reducing the time required for an LTP transfer module to move lipids from one membrane to the other, or because the lipid-transfer module had access to the two membranes. By contrast, our system is unique as Osh6 is neither attached to the membrane nor has a membrane tethering capacity, which is a function ensured by Ist2. We can thus demonstrate that mutations of Osh6 that impair its binding to the Ist2 IDR (Osh6-3M) reduce the PS transfer rate in otherwise intact synthetic ER-PM contact sites, implying that a closer proximity between membranes is not sufficient to confer a kinetic advantage. This aligns with our observations that Osh6-mediated PS transfer is strongly impaired in yeast expressing a mutant form of Ist2 unable to recruit Osh6 but still able to form ER-PM contact sites (*16*). A limitation of our reconstitution system is that we did not control the distance between membranes connected by the Ist2 IDR. This distance likely equals twice the hydrodynamic radius of the Ist2 IDR construct (5.5 nm; fig. S3G) and is shorter than the intermembrane gap in ER-PM contact sites. Nevertheless, by combining our in vitro and in vivo assays, we can conclude that Osh6 and, more generally, LTPs can function optimally at contact sites because they are concentrated in these substructures, not because they benefit from a short intermembrane space.

Our studies also provide structural insights into how Osh6 binds to Ist2 by experimentally validating models of the Osh6:Ist2 complex generated by AlphaFold3 (*50*). The algorithm identified the 727-to-750 segment in the full-length Ist2 IDR as the Osh6-binding motif and provided models in line with our previous results obtained by two-hybrid assays, pointing to the contribution of Osh6’s residues (D141 and L142) and Ist2’s residues (T736) to Osh6:Ist2 interaction (*16*). Moreover, these models allow us to identify several Osh6 residues (E170, Y391, Q417, and T420) that are engaged in H-bonds with Ist2 residues, as also indicated by the structures of Osh6 recently solved in complex with Ist2[732–761] and Ist2[732–757] peptides (*29*). We confirmed the relevance of these models by testing Osh6 mutants, showing that Q417 and E170 residues are critical for the Osh6:Ist2 interaction. Our data provide evidence that the Ist2-binding site on the Osh6 surface is distal to the entrance of the lipid-binding pocket, thereby explaining why Osh6 can transfer lipids when it binds to the Ist2 IDR. The β barrel that constitutes the core of the ORD, hosting lipid ligands, is a highly conserved structure among ORP/Osh proteins. Still, it is decorated, notably at its C terminus, by structural elements that strongly differ depending on ORP/Osh subfamilies (*6*). The fact that Osh6 associates with Ist2 mainly via its C-terminal region establishes that these structural elements allow for a specific interaction between an ORD and a partner. Whether this is true for other ORP/Osh proteins remains to be defined.

Recently, we and others have unveiled that the TM domain of Ist2 has scramblase activity (*28*, *29*). Thus, in addition to its role as an ER-PM tether and Osh6-binding partner, Ist2 equilibrates lipids between the leaflets of the ER membrane, suggesting possible coupling between transbilayer and intermembrane lipid transport. We tested this model in vitro, showing that Ist2, reconstituted in ER-like membranes, sustains the transfer of PS from these membranes to PM-like membranes by Osh6. This is likely because Ist2, via its scramblase activity, replenishes the PS pool at the surface of the ER-like membranes, which is accessible to Osh6. Therefore, we established that the coordinated work of a scramblase and an LTP can ensure lipid flux between a membrane’s inner leaflet and another membrane’s outer leaflet. Our first data do not suggest that Ist2 scrambling activity promotes Osh6-mediated PS transfer activity in yeast; however, one issue may be the sensitivity of the assays (*28*). It is also possible that the coupling between Ist2 and Osh6 observed in vitro might serve another function. It is assumed that scramblases could prevent the membranes from being destabilized following the delivery or extraction of lipids by LTPs, via the reequilibration of lipids between the membrane leaflets. Such a mechanism of compensation has been proposed for autophagosome formation, as LTP ATG2 can associate with the ER-resident scramblases TMEM41B/VMP1 but also the ATG9 scramblase present in the nascent autophagosome (*38*). Likewise, the LTP VPS13A and the scramblase XK, which are essential for PS exposure in adenosine 5′-triphosphate–stimulated cells and whose mutations cause pathological conditions with similar manifestations, have been shown to interact at ER-PM contacts (*70*, *71*). Therefore, one possibility is that Ist2, by transporting PS as well as other phospholipids (*28*) across the ER membrane, maintains the integrity and/or proper lipid composition of the two leaflets of this membrane when PS is delivered to the PM by Osh6.

Last, we do not yet know to what extent the coupling between the transport activities of Osh6 and Ist2 depends on their physical association and the capacity of Ist2 to connect membranes. We found that the scramblase activity of both full-length Ist2 and Ist2ΔC sustained Osh6-mediated PS transfer from PLs to PM-like liposomes. However, we could not detect faster PS transfer when using PLs prepared with Ist2, although the latter can tether Osh6 to PM-like membranes via its IDR. This could be due to a technical limitation of our transport assay using PLs, in which the concentration of Ist2 was estimated to be two orders of magnitude lower than that of Osh6, in contrast to the near-1:1 stoichiometry in assays with Osh6 and the Ist2 IDR. Further work combining in vitro assays with cellular investigations will be needed to fully understand how the lipid transport activities of Osh6 and Ist2 are coupled at ER-PM contact sites and how this coupling affects cellular function.

## MATERIALS AND METHODS

### Protein expression, labeling, and purification from *E. coli*

Osh4, Osh6, Osh6(noC/S190C), Osh6(H157A/H158A), other Osh6 mutants, ORD^ORP8^ and ORD^ORP8^ (H514A/H515A), and NBD-PH_PLCδ1_ were purified as previously described (*44*, *72*). To obtain Osh6 labeled with an ATTO590 maleimide (ATTO-TEC) or an Alexa Fluor 647-C2 maleimide dye (Invitrogen), an Osh6(noC/T262C) mutant was produced, labeled with the respective dye and purified following the protocol in (*45*). The concentration of all these constructs was determined by ultraviolet spectrophotometry.

GST-Ist2[590–936], GST-Ist2[590–946], GST-_C_Ist2[590–936], GST-_C_Ist2[590–946], GST-Ist2[590–946]Δ727–749, GST-Ist2[590–768], and GST-Ist2[727–776] were expressed in *Escherichia coli* (BL21-GOLD(DE3)) competent cells (Stratagene) grown in Luria Bertani Broth (LB) medium at 30°C overnight upon induction with 1 mM isopropyl β-d-1-thiogalactopyranoside. When the optical density of the bacterial suspension, measured at 600 nm (OD_600nm_), reached a value of 0.6 to 0.7, bacterial cells were harvested and resuspended in cold TN buffer (50 mM tris-HCl, pH 7.4, 150 mM NaCl, and 2 mM DTT) supplemented with 1 mM phenylmethylsulfonyl fluoride (PMSF), 10 μM bestatin, 1.6 μM pepstatin A, and cOmplete, EDTA-free protease inhibitor tablets (Roche). Cells were lysed in a Cell Disruptor TS SERIES (Constant Systems Ltd.), and the lysate was centrifuged at 186,000*g* for 1 hour and 30 min. Then, the supernatant was applied to Glutathione Sepharose 4B (Cytiva) for 3 hours and 30 min at 4°C. To purify the Ist2[590–936], _C_Ist2-[590–936], Ist2[590–946], and _C_Ist2[590–946] constructs, the beads were washed four times with TN buffer devoid of protease inhibitors; the beads were incubated with thrombin overnight at 4°C to cleave off each Ist2 construct from the GST domain. Each construct was recovered in the supernatant after several cycles of centrifugation and washing of the beads, concentrated, and then injected onto an XK-16/70 column packed with Sephacryl S-200 HR to be purified by size exclusion chromatography. The fractions with ~100% pure Ist2 construct were pooled, concentrated, and supplemented with 10% (v/v) pure glycerol (Sigma-Aldrich). Aliquots were prepared, flash-frozen in liquid nitrogen, and stored at −80°C. The protein concentration was determined using a bicinchoninic acid (BCA) assay. For some experiments, a volume of 100 μl from a stock solution of _C_Ist2[590–946] was applied onto a 0.5-ml Zeba spin desalting column [7-kDa molecular weight cutoff (MWCO)] equilibrated with freshly degassed 50 mM Hepes, pH 7.4, and 120 mM K-acetate (HK) buffer, according to the manufacturer’s instructions, to remove DTT from the protein and immediately used. To purify the GST-Ist2[590–946], GST-Ist2[590–946]Δ727–749, GST-Ist2[590–768], and GST-Ist2[727–776] constructs, the beads were washed four times with TN buffer devoid of protease inhibitors, and then the beads were incubated with TN buffer containing 20 mM glutathione and 2 mM DTT. The protein concentration was determined using a BCA assay.

ORD^Osh3^ (Osh3[605–996]) was produced in fusion with an N-terminal His-thioredoxin tag in *E. coli* cultured in ZYM autoinducible medium for 6 hours at 37°C and then overnight at 25°C. Bacterial cells were then lysed by sonication, and cell debris and insoluble materials were removed by centrifugation. The supernatant was loaded into a Hitrap column and equilibrated with 2× phosphate-buffered saline (buffer A) and 5% of 100 mM tris, pH 8.0, and 300 mM imidazole buffer (buffer B). The protein was eluted by increasing buffer B to 100% over 20 ml. After dialysis overnight against 20 mM tris, pH 8, and 150 mM NaCl buffer, the His-thioredoxin tag was cleaved off from the rest of the construct using HRV 3C protease. The protein was further purified by size exclusion chromatography using a 26/60 Superdex 75 column equilibrated with 20 mM tris, pH 8, and 150 mM NaCl buffer. The protein was concentrated, flash-frozen in liquid N_2_, and stored at −80°C. The protein concentration was determined using a BCA assay. Plasmids used for protein expression are listed in table S1.

### Production and purification of Ist2-3C-Bad from yeast

W303.1b/*GAL4* (*a*, *leu2-3*, *his3-11*, *trp1-1:TRP1-GAL10-GAL4*, *ura3-1*, *ade2-1*, *can r*, *cir +*) yeast strain was transformed by the lithium-acetate method. Yeast cultures and recombinant protein expression were performed as previously described (*73*). Yeast cells were then harvested by centrifugation, washed with ice-cold deionized H_2_O and then with ice-cold TEKS buffer (50 mM tris-HCl, pH 7.5, 1 mM EDTA, 0.1 M KCl, and 0.6 M sorbitol), and resuspended in TES buffer (50 mM tris-HCl, pH 7.5, 1 mM EDTA, and 0.6 M sorbitol) supplemented with protease inhibitors (SIGMAFAST EDTA-free protease inhibitor cocktail) and 1 mM PMSF. The cells were subsequently broken with 0.5-mm glass beads using a “Pulverisette 6” planetary mill (Fritsch). The crude extract was spun down at 1000*g* for 20 min at 4°C to remove cell debris and nuclei. The resulting supernatant was centrifuged at 20,000*g* for 20 min at 4°C, yielding the S2 supernatant and P2 pellet. The S2 supernatant was then ultracentrifuged at 125,000*g* for 1 hour at 4°C. The resulting P2 and P3 pellets were resuspended at 30 to 50 mg ml^−1^ of total protein in TES buffer.

To purify Ist2 and Ist2ΔC, membranes obtained after expression of Ist2 (P2 or P3) were diluted to 5 mg ml^−1^ of total protein in ice-cold buffer A [50 mM Mops-tris, pH 7.0, 500 mM NaCl, and 20% (w/v) glycerol], supplemented with 1 mM PMSF and protease inhibitors. *n*-Dodecyl-β-d-maltoside (DDM) was then added to 15 mg ml^−1^, resulting in a DDM/protein ratio of 3/1 (w/w). The suspension was then stirred gently on a wheel for 1 hour at 4°C. Insoluble material was pelleted by centrifugation at 100,000*g* for 1 hour at 4°C. The supernatant was applied onto streptavidin-sepharose resin (1 ml per 3 mg of Ist2) and incubated for 2 hours at 6°C to allow binding of BAD-tagged Ist2 to the resin. The resin was washed four times with three resin volumes of ice-cold buffer A supplemented with DDM (0.5 mg ml^−1^). Elution was performed by adding 70 μg of purified HRV-3C protease per milliliter of resin and overnight incubation at 6°C. Before reconstitution, the eluted fraction was concentrated to 0.3 to 0.4 mg ml^−1^ using a Vivaspin unit (100-kDa MWCO).

### Peptides

The Ist2[729–768] peptide (SYGVAGATLPETIPTSKNYYLRFDEDGKSIRDAKSSAESS) and its scrambled version (DKYASNSAKSTYGIRIPVATFESLLRSKSGETDAYGEDPS) were from Proteogenix. The peptide purity was >95%.

### Lipids

18:1/18:1-PC (DOPC), 16:0/18:1-PC (POPC), 16:0/18:1-PS (POPS), brain PI(4)P (l-α-phosphatidylinositol 4-phosphate), brain PI(4,5)P_2_ (l-α-phosphatidylinositol 4,5-bisphosphate), NBD-PE [1,2-dioleoyl-*sn*-glycero-3-phosphoethanolamine-*N*-(7-nitro-2-1,3-benzoxadiazol-4-yl)], Rhod-PE, 16:0/12:0 NBD-PS, and 18:1 MPB-PE were purchased from Avanti Polar Lipids.

### Liposome preparation

In glass tubes, lipids stored in CHCl_3_ or CHCl_3_/methanol stock solutions were mixed at the desired molar ratio. The tubes were prewarmed at 33°C for 5 min, then the solvent was dried under a nitrogen flux for 25 min, and the tubes were placed in a vacuum chamber for 40 min to remove the remaining solvent. The lipid film was hydrated in HK buffer or 50 mM Hepes, pH 7.4, and 210 mM sucrose buffer to obtain a suspension of multilamellar vesicles. The multilamellar vesicle suspensions were frozen, thawed five times, and then extruded through polycarbonate filters of 0.2 μm in pore size using a miniextruder (Avanti Polar Lipids). Liposomes were stored at 4°C and in the dark when containing fluorescent lipids and used within 2 days.

### PL preparation

Liposomes were formed from DOPC or a POPC/POPS (9:1, mol/mol) mixture. Lipids were dissolved at 10 mg ml^−1^ in 5 ml of CHCl_3_ and dried in a rotavapor for ~30 min under pressure to form a thin lipid film in a glass balloon. After CHCl_3_ evaporation, the lipid film was placed under vacuum in a desiccator for at least 1 hour to eliminate CHCl_3_ traces. Lipids were then suspended in 12.5 ml of buffer R (50 mM Mops-tris, pH 7, and 200 mM NaCl), yielding multilamellar vesicles at a final concentration of 4 mg ml^−1^. The vesicles were aliquoted, flash-frozen in liquid nitrogen, and stored at −80°C. Immediately before reconstitution, 400 μl of the vesicles was thawed and allowed to equilibrate at room temperature. All subsequent steps were performed at room temperature. The vesicles were then solubilized for 15 min with Triton X-100 (TX-100) under agitation using a magnetic stirrer. The detergent:lipid ratio used was 2.5:1 (w/w) as determined in (*74*), resulting in final concentrations of 7 mg of TX-100 per milliliter and 2.7 mg of lipids per milliliter. Then, 2 mol % of NBD-PS resuspended in DDM (0.5 mg ml^−1^) was added to the lipid/TX-100 mixture, along with 1 mM EGTA. The purified protein was added to the NBD-labeled detergent/lipid suspension at a 14.4 mg mmol^−1^ protein/lipid ratio (∼0.05 mg ml^−1^ protein) and incubated for 30 min at room temperature. TX-100 and DDM were removed by adding prewashed Bio-beads SM-2 adsorbent (Bio-Rad) in three steps: First, the sample was incubated for 2 hours with 10 mg of Bio-beads per milligram of TX-100, then a second aliquot of Bio-beads was added (10 mg/mg TX-100), and the sample was incubated for another 2 hours. Second, Bio-beads from the first two steps were removed, and an additional 20 mg of Bio-beads per milligram was added for 1 hour to the sample (*74*). Last, the PLs were separated from the beads and either stored at 4°C for up to 1 week or flash-frozen in liquid N_2_ and stored at −80°C.

### Preparation of the apo and lipid-bound forms of Osh6

For some experiments, Osh6 and its AF647-labeled version were prepared in an apo form or in a 1:1 complex with PS or PI(4)P. Protein (3.6 μM) was incubated with “heavy” liposomes (800 μM) composed of DOPC/POPS (95:5) or DOPC/brain PI(4)P (95:5), encapsulating 50 mM Hepes, pH 7.4, and 210 mM sucrose buffer, in a volume of 250 μl of HK buffer. The apo form of Osh6 was prepared by incubating the protein with pure DOPC liposomes. Each sample was mixed with liposomes by agitation for 30 min at 30°C and then centrifuged at 400,000*g* for 20 min at 20°C to pellet the liposomes using a fixed-angle rotor (Beckmann TLA 120.1). A fraction of each supernatant (200 μl) containing Osh6 loaded with lipid was collected, and the concentration of each complex was assessed by measuring sample absorbance at λ = 280 nm (ε = 55,810 M^−1^ cm^−1^) and, in the case of AF647-Osh6, at λ = 647 nm (ε = 265,000 M^−1^ cm^−1^).

### GST pull-down assay

A volume of 50 μl of Glutathione Sepharose 4B beads (slurry) was combined with either GST, GST-Ist2[590–946], GST-Ist2[590–768], GST-Ist2[727–776], or GST-Ist2[590–946]Δ727–749 (2.5 μM final concentration) in a total volume of 150 μl of buffer (50 mM tris, pH 7.4, 120 mM NaCl, 1 mM MgCl_2_, 1% TX-100, 1 mM DTT, 10% glycerol, and 0.25 mM PMSF). The samples were incubated for 30 min at 4°C under constant shaking (800 rpm). After washing the beads to remove the unbound GST construct, Osh6 or its mutated forms, Osh4, ORD^Osh3^, or ORD^ORP8^, was added at a concentration of 2.5 μM in a final volume of 150 μl buffer. Samples were incubated at 25°C for 1 hour under constant shaking. Last, the beads were washed, and the amount of bound proteins was analyzed by SDS–polyacrylamide gel electrophoresis (SDS-PAGE).

### Protein size measurement

The experiments were performed at 25°C using a Wyatt DynaPro-99-E-50 System (Protein Solutions). A small volume of each purified protein (200 μl) was dialyzed using a Slide-A-Lyzer MINI dialysis device (MWCO, 3.5 kDa) three times against HK buffer for 30 min to remove glycerol from protein stocks and to exchange buffer. Then, the samples were subjected to ultracentrifugation at 100,000*g* for 20 min at 20°C to pellet the protein aggregates. A volume of 20 μl of the supernatant was added to the DLS quartz cuvette. A set of 12 autocorrelation curves was acquired, and the data were analyzed using the regularization algorithm of Dynamics version 6.1 software.

### Circular dichroism

The experiments were performed on a Jasco J-815 spectrometer at room temperature with a quartz cell of 0.05 cm in path length (Starna Scientific Ltd.). Each protein was dialyzed in a Slide-A-Lyzer MINI dialysis device (MWCO, 3.5 kDa) three times against 20 mM tris, pH 7.4, and 120 mM NaF buffer for 30 min to remove glycerol or DTT from the protein stock and to exchange buffer. Then, the samples were subjected to ultracentrifugation at 100,000*g* for 20 min at 20°C to pellet protein aggregates, and the supernatant was collected. Each CD spectrum is the average of 10 scans recorded from λ = 190 to 260 nm with a bandwidth of 1 nm, a step size of 0.5 nm, and a scan speed of 50 nm min^−1^. The protein concentration was determined at λ = 280 nm by spectrophotometry or by densitometry after SDS-PAGE against a bovine serum albumin concentration range. A control spectrum of buffer was subtracted from each protein spectrum. The percentages of the protein secondary structure were estimated by analyzing their CD spectrum (in the 190- to 250-nm range) using the BeStSel method provided online (*75*).

### Microscale thermophoresis

The Ist2[729–768] peptide and its scrambled version were solubilized in 10 mM Hepes, pH 7.4, and 150 mM NaCl buffer supplemented with 0.5% Tween 20 and 3 mM EDTA (HBS-EP+ buffer) to prepare a stock solution. Ist2[590–936] was dialyzed in a Slide-A-Lyzer MINI dialysis device (MWCO, 3.5 kDa) three times against HBS-EP+ buffer for 30 min to remove glycerol and DTT from protein stocks and to exchange buffer. The concentration of peptides and Ist2[590–936] solutions was determined by spectrophotometry at λ = 280 nm. Then, a series of 16 1:1 dilution was prepared using HBS-EP+ buffer in a nonbinding 96-well black plate (Greiner Bio-One), producing ligand concentrations ranging from 10^−9^ to 10^−3^ M. Each ligand dilution (50 μl) was mixed with 10 μl of a stock solution of AF647-Osh6 (120 nM), which led to a final concentration of AF647-Osh6 of 20 nM. The plate was incubated for 10 min under constant shaking at 25°C. Each sample was loaded in Premium Monolith NT.115 Capillaries (NanoTemper Technologies). MST was measured using a Monolith NT.115 instrument (NanoTemper Technologies) at an ambient temperature of 25°C. Instrument parameters were adjusted to 50% light-emitting diode power and high MST power. Data from three independent measurements were fitted with a nonlinear regression model in GraphPad Prism using the signal from an MST on-time of 5 s to obtain a dissociation constant *K*_D_.

### CPM accessibility assay

On the day of the experiment, 100 μl from a stock solution of Osh6(noC/S190C) construct was applied onto a 0.5-ml Zeba spin desalting column (7-kDa MWCO) equilibrated with freshly degassed HK buffer, according to the manufacturer’s instructions, to remove DTT. The concentration of the eluted protein was determined by spectrophotometry, considering ε = 55,810 M^−1^ cm^−1^ at λ = 280 nm. A stock solution of CPM (Sigma-Aldrich) at 4 mg/ml was freshly prepared as described in (*76*) by mixing 1 mg of CPM powder in 250 μl of dimethyl sulfoxide. Thereafter, 50 μl of this solution was diluted in a final volume of 2 ml of HK buffer and incubated for 5 min at room temperature. The solution was protected from light and used immediately. In individual wells of a 96-well black plate (Greiner Bio-one), Osh6(noC/S190C) at 400 nM was incubated either with liposomes (400 μM total lipid) only made of DOPC or additionally containing 5% brain PI(4)P or POPS in 200 μl of HK buffer in the presence or absence of 40 μM Ist2[729–768] peptide for 10 min at 25°C under constant shaking. Then, a small volume of CPM stock solution was added to obtain a final concentration of 4 μM. After 25 min of incubation, fluorescence emission was measured at 465 nm (bandwidth, 5 nm) upon excitation at λ = 387 nm (bandwidth, 5 nm) using a fluorescence plate reader (TECAN M1000 Pro). Control intensities were recorded in the absence of protein for each condition.

### Flotation assay

The association of Ist2 constructs with membranes was measured by mixing the protein (0.75 μM) with liposomes of the desired composition, doped with 0.1 mol % of NBD-PE (750 μM lipids) for 1 hour at 25°C under constant shaking (800 rpm). For some experiments, this mixture was supplemented with 2 mM DTT before being mixed with Osh6 (0.75 μM) for 10 min under constant shaking. Next, in all cases, the liposome/protein mixture (final volume, 150 μl) was adjusted to 28% (w/w) sucrose by mixing 100 μl of a 60% (w/w) sucrose solution in HK buffer and overlaid with 200 μl of HK buffer containing 24% (w/w) sucrose and 50 μl of sucrose-free HK buffer. The sample was centrifuged at 201,600*g* (average centrifuge force) in a swing rotor (TLS 55 Beckmann) for 70 min. The bottom (250 μl), middle (140 μl), and top (110 μl) fractions were collected. The bottom and top fractions were analyzed by SDS-PAGE after staining with SYPRO Orange using a FUSION FX fluorescence imaging system.

### Aggregation assays

The experiments were performed at 25°C using a Wyatt DynaPro-99-E-50 System (Protein Solutions). L_ER_ liposomes (50 μM total lipids) composed of DOPC/MPB-PE/NBD-PE (89.9:10:0.1) or DOPC/NBD-PE (99.9:0.1) were mixed with an equivalent amount of L_PM_ liposomes composed of POPC/POPS/PI(4,5)P_2_/NBD-PE (64.9:30:5:0.1) or POPC/POPS/NBD-PE (69.9:30:0.1) in 20 μl of freshly degassed HK buffer and added to the quartz cell. A first set of 12 autocorrelation curves was acquired to measure the size distribution of the initial liposome suspension. Then, the _C_Ist2[590–946] construct (500 nM final concentration) was added manually and mixed thoroughly. For all the experiments, aggregation kinetics were measured by acquiring one autocorrelation curve every 10 s for 80 min. At the end of the experiment, a set of 12 autocorrelation functions was acquired. The data were analyzed using two different algorithms provided by Dynamics version 6.1 software (Protein Solutions). The autocorrelation functions were fitted during the kinetics, assuming that the size distribution is a simple Gaussian function. This mode, called the monomodal or cumulant algorithm, gives a mean hydrodynamic radius, *R*_H_, and width (or polydispersity). The polydispersity is represented in the kinetics measurements by the shaded area. It can reach tremendous values because of the simultaneous presence of free liposomes and liposome aggregates of various sizes. The autocorrelation functions were fitted before and after the aggregation process using a regularization algorithm that can resolve several populations of different sizes, such as free liposomes and liposome aggregates.

### Confocal microscopy with liposomes

Giant liposomes were produced by polyvinyl alcohol (PVA)–assisted swelling (*77*). Fifty microliters of 5% (w/w) PVA solution (average molecular weight, 146,000 to 186,000; Sigma-Aldrich) was spread on a glass coverslip (diameter, 1.5 mm) and dried at 55°C for 30 min in an oven. Then, a volume of 20 μl of lipid mixture (1 mg/ml) with the desired lipid ratio in chloroform was spread on the top of the PVA layer. The solvent was allowed to evaporate overnight in a vacuum chamber. A volume of 200 μl of swelling buffer (20 mM Hepes, pH 7.5, and 210 mM sucrose) was added to the coverslip to form giant liposomes. After 20 min of incubation at room temperature, vesicles (~130 μM total lipids) were collected by pipetting and kept at room temperature, protected from light. A volume of 10 μl of liposome suspension (2 mM lipids) was gently added to 50 μl of the suspension of giant liposomes. Then, a volume of 3.5 μl of a stock solution of DTT-free _C_Ist2[590–946] (28 μM) was gently added. After 30 min, the liposomes were imaged in μ-Slide 18 well Ibitreat (ibidi); wells were previously coated for 1 hour with bovine serum albumin (2 mg/ml) and washed three times with degassed HK buffer. A volume of 5 μl of the mixture was added to 100 μl of HK buffer in each well for imaging. For the experiments with AF647-Osh6, a volume of 0.5 μl of the fluorescent protein (stock solution, 18 μM) was previously added to the well. After a few minutes, the samples were observed at room temperature using a Leica TCS SP8 STED 3X in confocal mode. Images were acquired through a 63×/1.4–numerical aperture (NA) oil objective using LAS X software and analyzed using ImageJ software.

### NBD-PS transfer assays

These assays were performed using a Jasco FP-8300 spectrofluorometer at 30°C with Osh6 and ORD^ORP8^. For experiments with three liposome populations, L_ER_ liposomes (200 μM lipids) composed of DOPC/MPB-PE/NBD-PS (88:10:2) were mixed with an equivalent amount of L_PM_ liposomes composed of POPC/POPS/PI(4,5)P_2_/Rhod-PE (63:30:5:2) and L_O_ liposomes composed of POPC/POPS (70:30) in HK buffer. Then, a small volume of DTT-free _C_Ist2[590–946] (500 nM final concentration) or HK buffer was mixed for 10 min with the liposomes. The capacity of free MPB-PE lipids to form covalent bonds with thiol-containing proteins was then neutralized by adding 2 mM DTT. Upon adding Osh6 or ORD^ORP8^ (200 nM), the specific transfer of NBD-PS to L_PM_ liposomes was measured for 11 min by following FRET between NBD-PS and Rhod-PE (λ_em_ = 580 nm; bandwidth, 2.5 nm) on excitation at λ_ex_ = 460 nm (bandwidth, 2.5 nm). Similar experiments were performed with L_PM_ liposomes composed of POPC/POPS/PI(4,5)P_2_ (65:30:5) and L_O_ liposomes composed of POPC/POPS/Rhod-PE (68:30:2). The signal (*F*) measured over time was normalized to the averaged signal measured just before the injection of Osh6 (*F*_0_). The NBD-PS transfer rate was quantified by determining the time required to achieve half of PS equilibration between L_ER_ and L_PM_, or between L_ER_ and L_O_ liposomes after Osh6 injection, and calculating a rate corresponding to 1/*t*_1/2_ (respectively, ER → PM and ER → O transfer rates). An Ist2-dependent $\frac{\text{ER}\to \text{PM}}{\text{ER}\to O}$ transfer rate ratio (*R*_Ist2_) was determined by dividing the $\frac{\text{ER}\to \text{PM}}{\text{ER}\to O}$ transfer rate ratio determined from kinetics measured in the presence of _C_Ist2[590–946] by the $\frac{\text{ER}\to \text{PM}}{\text{ER}\to O}$ ratio determined from kinetics measured in the absence of this construct. In addition, some kinetics were normalized to determine the amount of NBD-PS transferred to L_PM_ and L_O_ membranes. For this, measurements were performed with L_ER_ liposomes (200 μM lipids) composed of DOPC/MPB-PE/NBD-PS (89:10:1) mixed with an equivalent amount of L_PM_ liposomes composed of POPC/POPS/PI(4,5)P_2_/Rhod-PE/NBD-PS (62:30:5:2:1) and L_O_ liposomes composed of POPC/POPS (70:30) to determine *F*_max_, a FRET signal that would be measured if the entire pool of NBD-PS initially present in the outer leaflet of L_ER_ liposomes (2 μM) was transferred to L_PM_ liposomes. The amount of transferred NBD-PS by Osh6 in the different kinetics was then determined by considering that [PS] = 2 × *F*_Norm_, with *F*_Norm_ = (*F* − *F*_0_)/(*F*_max_ − *F*_0_).

For measuring PS transfer from PLs to liposomes, an 8-μl aliquot of PLs composed of DOPC/NBD-PS (98:2), containing Ist2, Ist2ΔC, or no protein (“mock”), was diluted in 570 μl of HK buffer in the cuvette thermostated at 25°C under constant stirring. The fluorescence of the NBD signal was identical under each condition. After 400 s of incubation, L_PM_ liposomes (50 μM lipids) made of POPC/POPS/PI(4,5)P_2_/Rhod-PE (63:30:5:2) were added. Five minutes later, Osh6(HH/AA) was injected, and the transfer of NBD-PS to L_PM_ liposomes was measured by following FRET between NBD-PS and Rhod-PE (λ_em_ = 580 nm; bandwidth, 2.5 nm) on excitation at λ_ex_ = 460 nm (bandwidth, 2.5 nm). The average signal (*F*_0_) measured before adding Osh6 was subtracted from the *F* value measured over time after adding Osh6. We also estimated the amount of NBD-PS that could be transported if all the molecules of NBD-PS from both the inner and outer leaflets of the PLs were accessible by recording reference kinetics with twice the amount of “mock” PLs. The averaged *F* measured at the end of the kinetics (*F*_max_), from which *F*_0_ was subtracted, was used to estimate the percentage of NBD-PS transferred from PLs to L_PM_ liposomes by Osh6 from the other kinetics.

### PI(4,5)P_2_ transfer assay

This assay was carried out using a Jasco FP-8300 spectrofluorometer. A suspension (540 μl) of L_A_ liposomes (200 μM total lipid) composed of DOPC/PI(4,5)P_2_/Rhod-PE (93:5:2) was mixed with 250 nM NBD-PH_PLCδ1_ in HK buffer. A cylindrical quartz cuvette was filled with this mixture; it was continuously stirred with a small magnetic bar, and thermostated at 30°C. After 2 min, 60 μl of a suspension of L_B_ liposomes (200 μM lipids), only composed of DOPC, was injected. Osh6 or its HH/AA mutant (250 nM) was injected 2 min later. The signal was followed by measuring the NBD fluorescence emission at λ = 530 nm (bandwidth, 5 nm) upon excitation at λ = 460 nm (bandwidth, 5 nm) with a time resolution of 1 s. The amount of PI(4,5)P_2_ transferred from L_A_ to L_B_ liposomes was determined considering that [PI(4,5)P_2_] = 2.5 × *F*_Norm_, with *F*_Norm_ = (*F* − *F*_0_)/(*F*_Eq_ − *F*_0_). *F* corresponds to the data point recorded over time, *F*_0_ is the average signal measured before the addition of Osh6, and *F*_Eq_ is the average signal measured in the presence of L_A-Eq_ and L_B-Eq_ liposomes containing 2.5 mol % PI(4,5)P_2_. At equilibrium, it is considered that one half of accessible PI(4,5)P_2_ molecules contained in the outer leaflet of L_A_ liposomes (i.e., corresponding to 5% of 0.5 × 200 μM total lipids) have been transferred into L_B_ liposomes.

### Scramblase assay

An 8-μl aliquot of either “mock” or Ist2-containing PLs was diluted to a final HK buffer volume of 600 μl. Then, the fluorescence of NBD-PS was monitored over time in a quartz cuvette using excitation at 460 nm (bandwidth, 2.5 nm) and emission at 534 nm (bandwidth, 2.5 nm). After 5 min, 6 μl of a stock solution of sodium dithionite (from a 1 M dithionite stock solution, stored at −20°C) was added (final concentration, 10 mM), and the quenching of the NBD signal was measured for 5 min. For the experiments with Osh6(HH/AA), 200 nM protein was added 260 s before adding dithionite. Kinetics have been analyzed and/or normalized using the following formula: (*F* − *F*_end_)/(*F*_start_ − *F*_end_) × 100, where *F* is the intensity measured over time, *F*_start_ is the intensity just before dithionite addition, and *F*_end_ is the intensity after TX-100 addition (near 0), which allows to determine the extent of NBD reduction by dithionite.

### Iodide collisional quenching

A volume of 10 μl of liposomes (mock) or Ist2-containing PLs was diluted in 50 mM Mops-tris, pH 7, and 40 mM Na_2_S_2_O_3_ buffer, directly in a quartz cuvette. Na_2_S_2_O_3_ was used to stabilize iodide ions in the solution. NBD fluorescence was followed by setting excitation and emission wavelengths at 470 and 530 nm, respectively, with slits of 5 nm, on a Horiba Jobin Yvon Fluorolog fluorimeter. The initial fluorescence (*F*_0_) was recorded for about 100 s, and then concentrations of KI ranging from 0 to 0.2 M were added. KCl was used to adjust the ionic strength to 0.2 M for each cuvette before adding samples and KI. The mean fluorescence (*F*) value recorded over 240 to 290 s after KI addition was taken and used to plot Δ*F* (*F*_0_ − *F*). The data were analyzed according to a modified Stern-Volmer equation (*78*, *79*): *F*_0_/Δ*F* = (1/fa·*K*[Q]) + (1/fa) where *F*_0_ is the fluorescence intensity in the absence of the quencher, Δ*F* is the fluorescence intensity in the presence of the quencher at concentration [Q] subtracted from *F*_0_, fa represents the fraction of fluorescence accessible to iodide ions, and *K* is the Stern-Volmer quenching constant.

### Modeling

Models of the Osh6:Ist2 IDR heterodimer were obtained with AlphaFold3 (*50*) using *S. cerevisiae* Osh6 and Ist2[590–946] sequences as input (UniProt: Q02201 and P38250, respectively). The identification of interchain H-bonds in the different models was done using PyMOL (https://pymol.org/).

### Plasmid, strains, and growth conditions for yeast experiments

Plasmids used for the yeast experiments are listed in table S2. The NEBuilder Hifi DNA Assembly Kit was used to generate the following plasmids: pOsh6-mCherry-E170A, pOsh6-mCherry-3M, and pIst2 derivative plasmids ([1–877], [590–946], [1–877]PM, short tail, short tail +23aa, short tail +40aa, very long tail, BS ER, and BS PM) according to the supplier’s instructions. A site-directed mutagenesis kit (Agilent) was used to generate the deletion of the 736-to-743 region from the pIst2 plasmid. Briefly, the plasmid was amplified using oligonucleotides excluding the 736-to-743 region. All constructs were verified by DNA sequencing.

Yeast strains are listed in table S3. Yeasts were grown in rich medium (yeast extract, peptone, and dextrose) or in synthetic dextrose (SD) medium containing 2% (w/v) glucose and appropriate amino acid dropout mix (MP Biomedicals). Yeast was transformed using the standard lithium acetate/polyethylene glycol procedure. SD medium was supplemented with 1 mM ethanolamine when growing *cho1*-deficient strains or *psd1*Δ*ist2*Δ. The *ist2*∆ *OSH6-GFP TCB1-TagRFP* strain was constructed by homologous recombination using the appropriate marker as indicated in the table.

### Live yeast cell imaging

Yeast cells were grown for 14 to 18 hours in appropriate SD medium at 30°C. Cells were diluted and harvested by centrifugation in the midlogarithmic phase (OD_600nm_ = 0.6 to 0.8) and prepared for visualization on glass slides, except for time-course experiments in which cells were maintained in a microfluidics chamber (see below). Imaging was conducted at room temperature using a Spinning disk SR system (Olympus), equipped with an oil immersion plan apochromat 60× objective (NA, 1.42), an sCMOS Fusion BT camera (Hamamatsu), and a spinning-disk confocal system CSU-W1 (Yokogawa) except for cellular PS transport assay (see below). BFP-, GFP-, and mCherry/tagRFP–tagged proteins were visualized with DAPI (4′,6-diamidino-2-phenylindole; 447/50), GFP (525/50), and mCherry (593/40) filters, respectively. Cells were imaged in 5 to 10 *z*-sections separated by 0.25 μm. Images were acquired using CellSens software (Olympus) and processed with Fiji (ImageJ).

### Cellular PS transport assay

Transport of PS in *cho1*Δ cells was performed as described previously (*8*, *48*). Briefly, 18:1 lyso-PS (Avanti Polar Lipids) was dried under argon and resuspended in SD medium to 54 μM lyso-PS and used within 1 to 3 hours after preparation. The PS transport assay was carried out using a Microfluidic Perfusion Platform (ONIX) driven by the interface software ONIX-FG-SW (Millipore). Strains *cho1*Δ and *cho1*Δ *ist2*^*736–743*Δ^, transformed with pC2_Lact_-GFP (*80*) and other plasmids, as indicated, were grown to OD_600nm_ = 0.6 to 0.8, injected into a YO4C microfluidics chamber, and maintained in a uniform focal plane. When imagining two strains simultaneously, we stained one strain with the vacuolar dye CMAC (Life Technologies) at 100 μM for 10 min and washed twice before mixing the two strains at a 1:1 ratio Normal growth conditions were maintained by flowing cells with SD medium or SD medium containing lyso-PS at 20.7 kPa (3psi). Cells were imaged every 2 min over 30 to 40 min in five *z*-sections separated by 0.7 μm. Imaging was performed at room temperature using an Axio Observer Z1 microscope (Zeiss) equipped with an oil immersion plan apochromat 100× objective (NA, 1.4), an sCMOS PRIME 95 camera (Photometrics), and a spinning-disk confocal system CSU-X1 (Yokogawa). GFP-tagged proteins and CMAC/BFP–tagged proteins were visualized with a GFP Filter 535AF45 and a DAPI Filter 450QM60, respectively. Images were acquired with MetaMorph 7 software (Molecular Devices).

### Yeast image analysis

Images were processed with Fiji (ImageJ). Quantification of Osh6-mCherry localization was performed manually from a stack of 10 *z-*sections. Distribution of C2_Lact_-GFP was analyzed as described previously (*8*, *48*). Briefly, a three-dimensional stack of selected central *z*-sections over the entire time course was constructed manually. Quantification of peripheral and internal peaks was performed by profiling cell signal intensity across a transversal line drawn in each cell at the start of the time course; the external limit of the cell (perimeter) was selected, and total cell fluorescence was measured. Intensities of the peaks were quantified and normalized relative to the total signal. The cell profiles (peripheral and internal peaks) were followed and quantified over the time of the experiment. Data were processed in Excel and plotted using GraphPad.

### Yeast kinetic assays

The *psd1*Δ*ist2*Δ strains transformed with plasmids expressing BFP-Ist2 variants were grown for 14 to 18 hours at 30°C in SD-His medium supplemented with 2 mM ethanolamine. Cells were adjusted to an OD_600nm_ = 0.1 and grown in technical duplicates, in the presence or absence of ethanolamine, in 96-well microplates using the Spark microplate reader (Tecan). For each kinetic cycle, with an interval of 10 min, the cultures were shaken for 9 min with an amplitude of 2 mm and a frequency of 150 rpm, followed by a 1-min settling period before measuring the absorbance at 600 nm. Kinetic measurements continued until all strains reached the plateau of the stationary phase.

### Statistical analysis

Statistical analyses were performed using Prism (GraphPad). *P* values <0.05, <0.01, and <0.001 are identified with one, two, and three asterisks, respectively. ns, *P* ≥ 0.05. The number of replicates (*n*) used for calculating statistics is specified in the figure legends.
