# Supplementary material for: Ist2 promotes lipid transfer by Osh6 via its membrane tethering and lipid scramblase activities
Source: Sci Adv. 2025 Nov 14;11(46):eadz2217. doi: 10.1126/sciadv.adz2217 (PMC12617493; doi:10.1126/sciadv.adz2217)
Supplement: Supplementary file 1 — Figs. S1 to S8 Tables S1 to S3 Legend for data S1 [file sciadv.adz2217_sm.pdf]

Science Advances
AAAS

Supplementary Materials for

Ist2 promotes lipid transfer by Osh6 via its membrane tethering and lipid scramblase activities

Alicia Fabbre *et al.*

Corresponding author: Guillaume Drin, drin@ipmc.cnrs.fr

Sci. Adv. **11**, eadz2217 (2025)
DOI: 10.1126/sciadv.adz2217

The PDF file includes:

Figs. S1 to S8
Tables S1 to S3
Legend for data S1

Other Supplementary Material for this manuscript includes the following:

Data S1

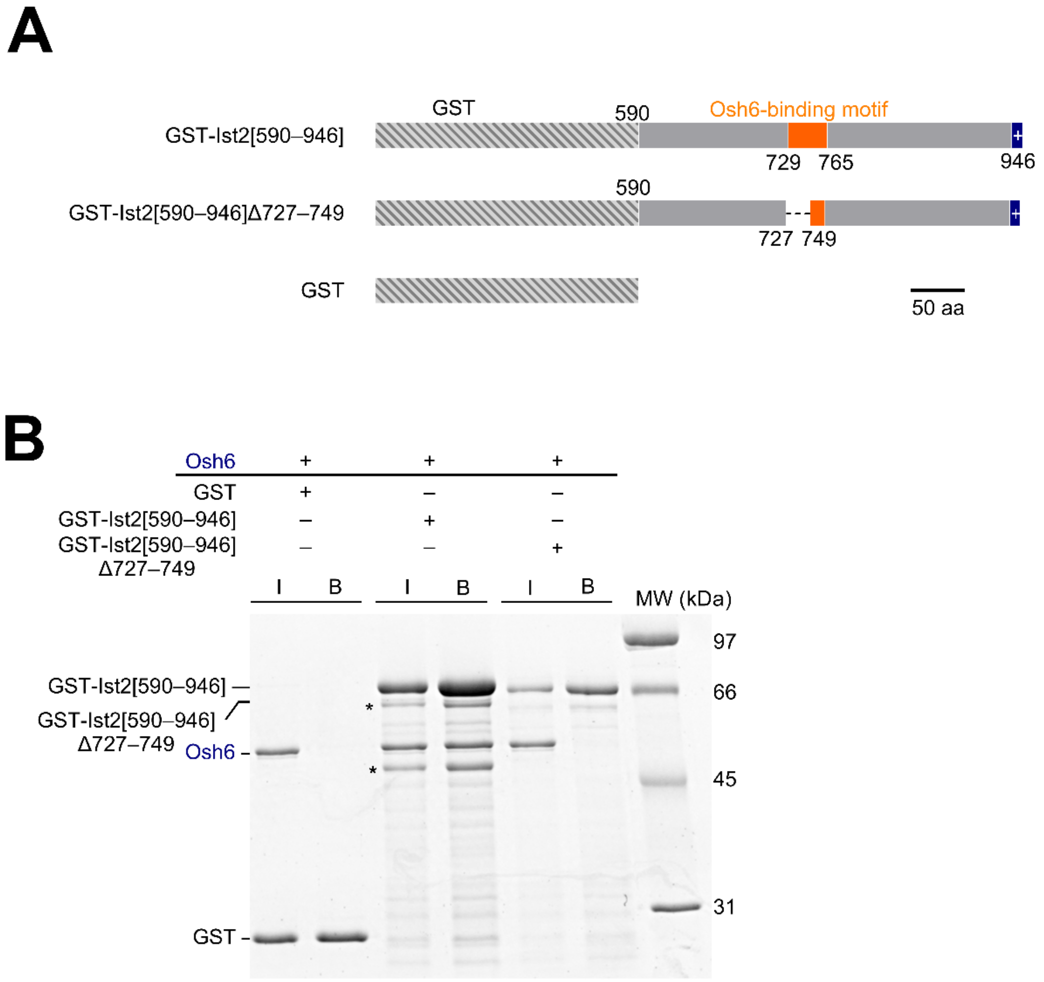

Fig. S1. Additional GST pull-down assays with Osh6.
Osh6 does not bind to the Ist2 IDR lacking a complete Osh6-binding motif. Purified GST, GST-Ist2[590–946], and GST-Ist2[590–946]Δ727–749 were immobilized on glutathione beads and incubated with Osh6. The arrows indicate the different GST-Ist2 constructs, and the stars indicate the main contaminants.

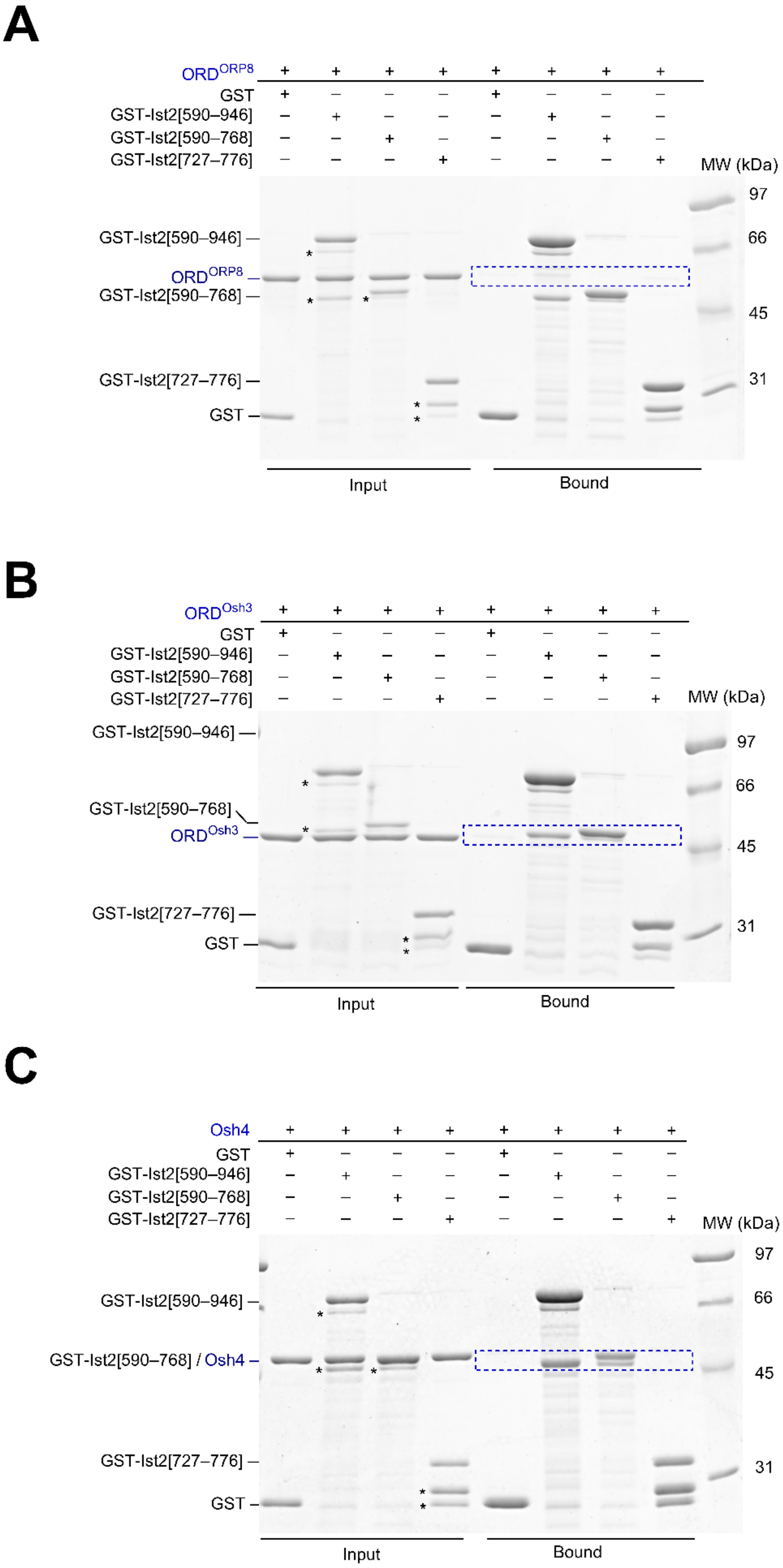
A
ORD^ORP8
GST
GST-Ist2[590–946]
GST-Ist2[590–768]
GST-Ist2[727–776]
MW (kDa)
97
66
45
31
GST-Ist2[590–946]
ORD^ORP8
GST-Ist2[590–768]
GST-Ist2[727–776]
GST
Input
Bound
B
ORD^Osh3
GST
GST-Ist2[590–946]
GST-Ist2[590–768]
GST-Ist2[727–776]
MW (kDa)
97
66
45
31
GST-Ist2[590–946]
GST-Ist2[590–768]
ORD^Osh3
GST-Ist2[727–776]
GST
Input
Bound
C
Osh4
GST
GST-Ist2[590–946]
GST-Ist2[590–768]
GST-Ist2[727–776]
MW (kDa)
97
66
45
31
GST-Ist2[590–946]
GST-Ist2[590–768] / Osh4
GST-Ist2[727–776]
GST
Input
Bound

Fig. S2. Additional GST pull-down assays with ORD^{ORP8}, $ORD^{Osh3,}$ and Osh4

Control experiments showed that **(A)** ORD^{ORP8}, **(B)** $ORD^{Osh3,}$ and **(C)** Osh4 do not bind to the Ist2 IDR. The experiments were performed as in Fig. 1A. Purified recombinant GST-Ist2[590–946], GST-Ist2[590–768], and GST-Ist2[727–776] constructs were immobilized on glutathione beads and incubated with ORD^{ORP8}, $ORD^{Osh3,}$ or Osh4. Input and bound fractions were analyzed using SDS-PAGE with SYPRO Orange staining. The arrows indicate the different GST-Ist2 constructs, and the stars indicate the main contaminants.

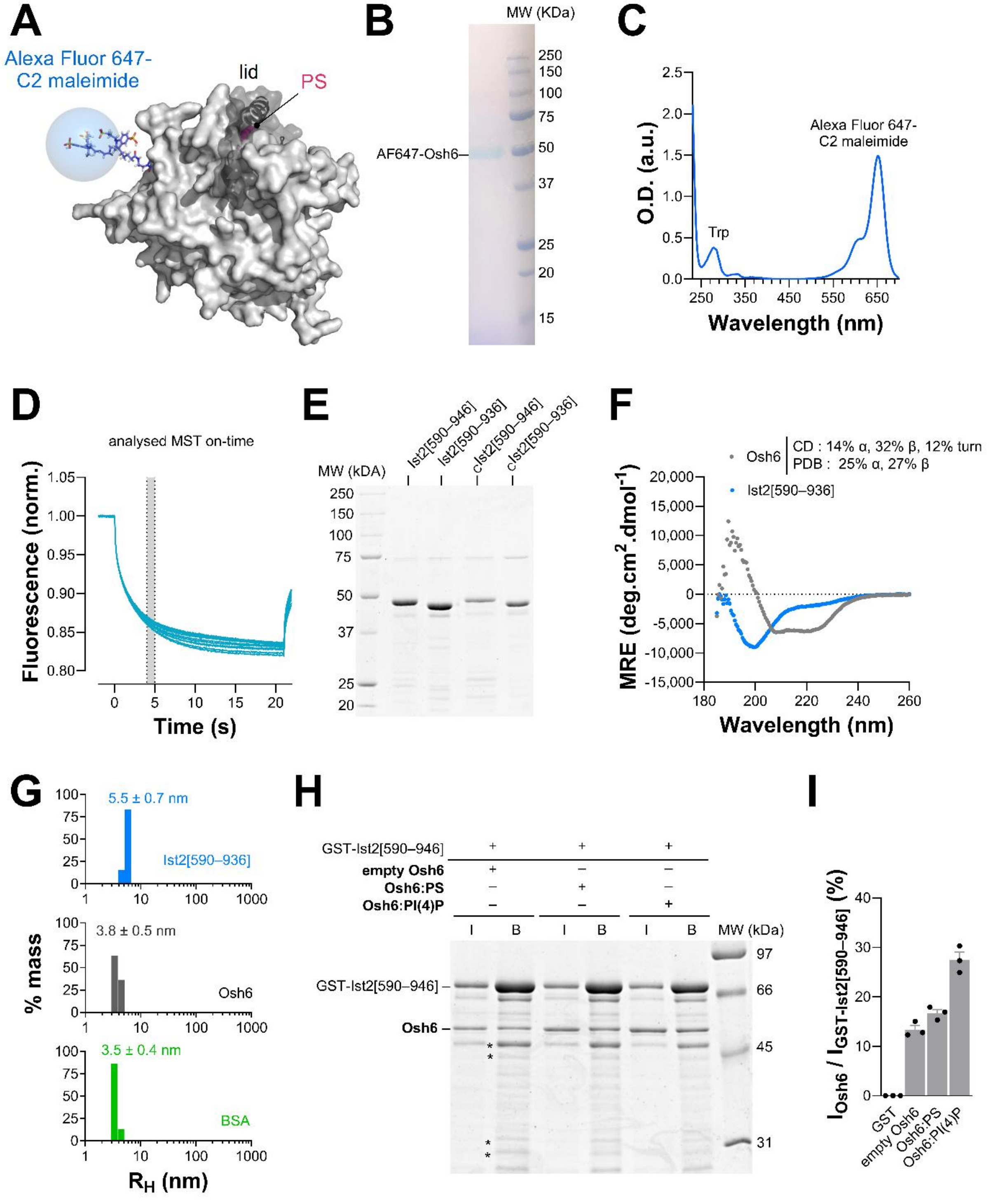

Fig. S3. Measurement of Osh6:Ist2 binding affinity

(A) Three-dimensional model of AF647-Osh6 based on the crystal structure of Osh6 (PDB ID: 4B2Z). The solvent-exposed cysteines C62, C162, and C389 are mutated to serines; a cysteine

replaces threonine T262. An Alexa Fluor 647-C2 maleimide moiety (in stick, with carbon in blue light, nitrogen in dark blue, and oxygen in red) is grafted to the thiol function of the C262 residue. The figure was prepared using PyMOL (http://pymol.org/).

(B) Analysis by SDS-PAGE of purified AF647-Osh6. The in-gel fluorescence was directly visualized without any staining. Lane 1: AF647-Osh6, lane 2: Precision Plus Protein™ All Blue Prestained Protein Standards (Bio-Rad).

(C) UV-visible absorption spectrum of AF647-Osh6. Considering a purity grade of 100% for the protein, the optical density value at 280 nm (Trp) and 650 nm (Alexa Fluor 647-C2 maleimide) indicated that Osh6 was labelled with the dye in a 1:1 ratio.

(D) Typical MST-based binding assay. The thermophoretic movement of AF647-Osh6 changes upon binding to the Ist2[729–768] peptide.

(E) SDS-PAGE of purified Ist2[590–936] and Ist2[590–946] constructs with or without an extra N-terminal cysteine.

(F) Far-UV CD spectrum of purified Ist2[590–936] (17.7 µM) and Osh6 (12.8 µM) in 20 mM Tris, pH 7.4, 120 mM NaF buffer at room temperature. The percentages of α-helix, β-sheet, and turn, derived from the analysis of the spectrum of Osh6, are given, as well as the values derived from the crystal structure (PDB ID: 4B2Z) using the DSSP algorithm. MRE, mean residue ellipticity.

(G) Hydrodynamic radius distribution by mass for Ist2[590–936] (35 µM; 37 kDa), Osh6 (50 µM, 51.6 kDa), and BSA (30 µM, 66.5 kDa) measured in HK buffer at 25 °C.

(H) GST pull-down with Osh6 in an empty or lipid-loaded state. Osh6 was mixed with liposomes made of DOPC or liposomes additionally containing 5 mol% POPS or brain PI(4)P, and separated from these liposomes by ultracentrifugation. Each form of the protein was incubated with GST-Ist2[590–946] immobilized on beads. Input (I) and bound (B) fractions were analyzed using SDS-PAGE.

(I) Quantification of the GST-pull down assays as shown in (H).

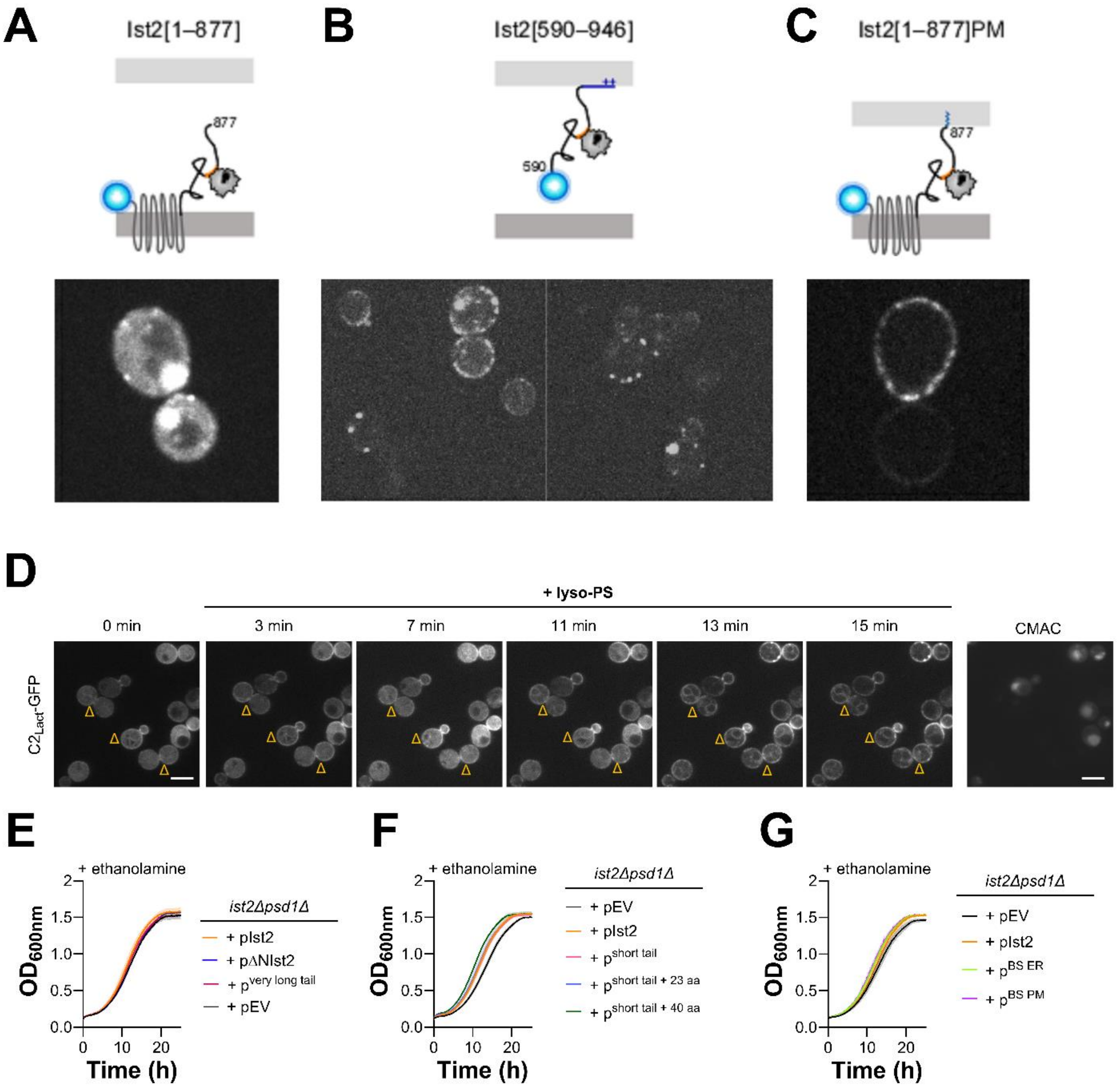

Fig. S4. Supplementary information for Fig. 2: Localization and functionality of Ist2 truncation mutants in yeast.

(A) Diagram of BFP-Ist2[1–877], lacking the PM binding region, and its localization in *ist2Δ* cells (lacking endogenous Ist2) (bottom panel). The bright spots likely represent BFP-Ist2[1–877] aggregates. This construct contains the full ER-embedded TM domain [1–589] and the cytosolic tail [590–878], with the Osh6-binding site depicted in orange, and Osh6 in grey. BFP is shown as a blue sphere.

(B) Diagram of BFP-Ist2[590–946], lacking the full TM domain, and its localization in *ist2Δ* cells (bottom panels, showing two fields of cells). Note the low fluorescent signal; the puncta likely represent BFP-Ist2[590–946] aggregates. The PM-binding polybasic region is shown in blue.

(C) Diagram of BFP-Ist2[1–877]PM, where the PM binding region is replaced by the CAAX prenylation motif that allows attachment of the IDR to the PM via the lipid anchor (shown in green).

Bottom panel shows that this construct localizes to the cortex of *ist2Δ* cells, and colocalizes with Osh6-GFP (not shown).

(D) Snapshots of the cellular PS transport assay, showing the localization of the PS-probe $C2_{Lact}$-GFP over time after the addition of lyso-PS. A mix of two strains was imaged in this experiment, *cho1Δ* and *cho1Δist2*$^{736–743Δ}$ (marked with "Δ" in the images); the *cho1Δ* strain was stained with the vacuolar dye CMAC (right-most panel) before the experiment to distinguish between the two strains. Quantification of this experiment is shown in Fig. 2C.

(E) Control growth curves of *ist2Δ psd1Δ* cells transformed with different pBFP-Ist2 plasmids, as indicated, where pEV represents an empty control plasmid. Yeast growth was monitored over time in minimal medium in the presence of ethanolamine at 30 °C.

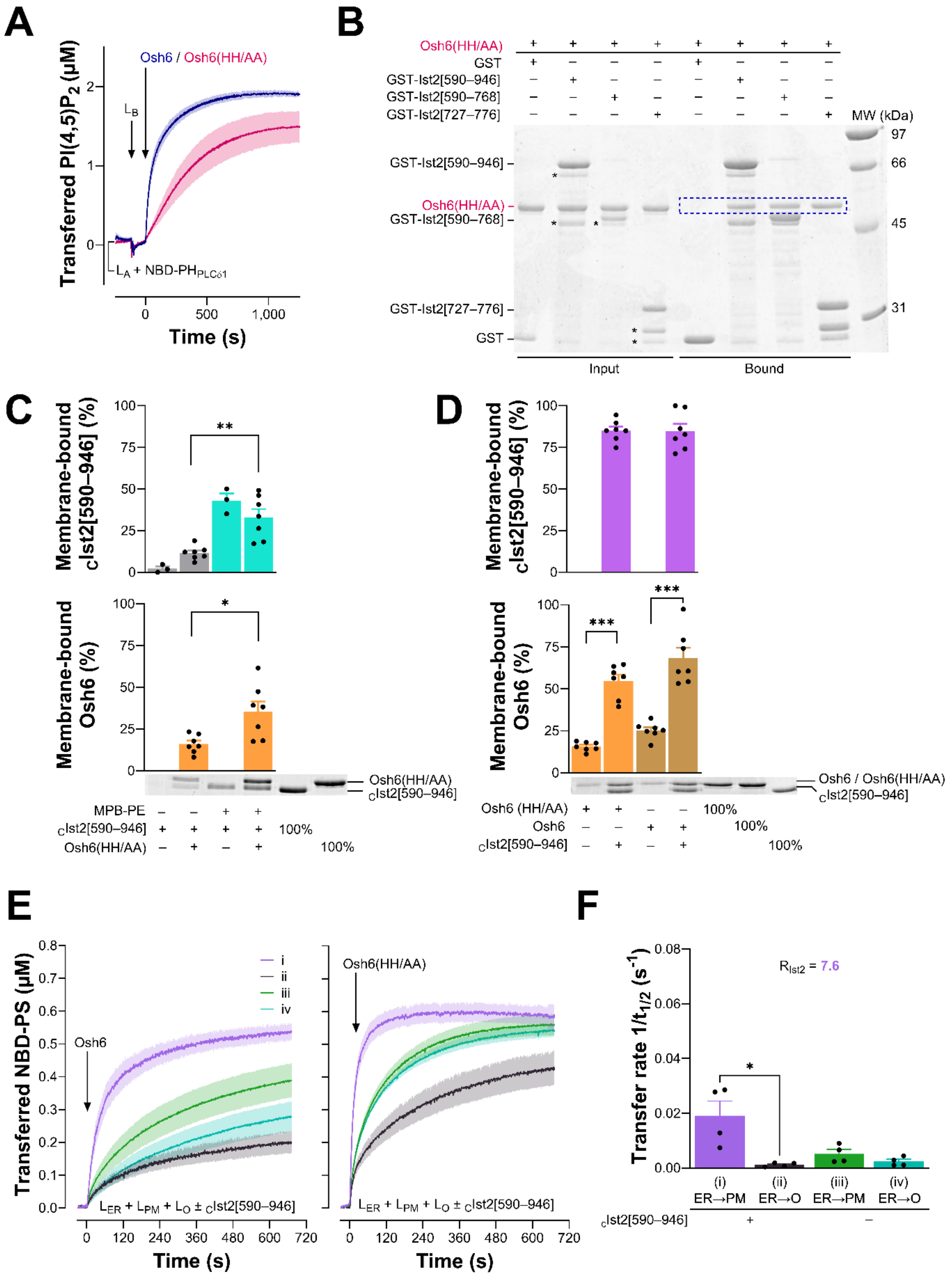
A
Transferred PI(4,5)P2 (µM)
Osh6 / Osh6(HH/AA)
LB
LA + NBD-PHPLCδ1
Time (s)
B
Osh6(HH/AA)
GST
GST-Ist2[590–946]
GST-Ist2[590–768]
GST-Ist2[727–776]
MW (kDa)
Input
Bound
C
Membrane-bound cIst2[590–946] (%)
Membrane-bound Osh6 (%)
MPB-PE
cIst2[590–946]
Osh6(HH/AA)
100%
D
Osh6 / Osh6(HH/AA)
Osh6 (HH/AA)
Osh6
E
Transferred NBD-PS (µM)
Osh6
Osh6(HH/AA)
LER + LPM + LO ± cIst2[590–946]
Time (s)
F
Transfer rate 1/t1/2 (s-1)
RIst2 = 7.6
(i) ER→PM
(ii) ER→O
(iii) ER→PM
(iv) ER→O

Fig. S5. Lipid transfer activities of Osh6 and Osh6(HH/AA) and interaction with the Ist2 IDR

(A) $PI(4,5)P_2$ transfer activity of Osh6 and Osh6(HH/AA). L_A liposomes (200 µM total lipids) composed of DOPC/$PI(4,5)P_2$/Rhod-PE (93:5:2) were mixed with NBD-$PH_{PLC\delta1}$ (250 nM) at 30 °C. After 2 min, DOPC liposomes (200 µM, L_B) were added. Two minutes later, Osh6 or Osh6(HH/AA) was injected (250 nM). The signal was normalized in terms of transferred $PI(4,5)P_2$. Each curve is the mean ± SEM of independent kinetics traces (n = 3).

(B) GST pull-down. GST-tagged segments of the Ist2 IDR were immobilized on glutathione beads and incubated with Osh6(HH/AA). Input and bound fractions were analyzed by SDS-PAGE. The stars indicate the main contaminants in GST-tagged construct preparation. As pointed out by the blue box, Osh6(HH/AA) interacts with the three GST-tagged segments of Ist2 IDR. No interaction was seen with GST alone.

(C) Flotation assay. $_C$Ist2[590–946] construct (0.75 µM) was mixed for 1 h with DOPC liposomes, containing or not 10% MPB-PE, and doped with 0.1% NBD-PE. Then, DTT (1 mM) was added to stop the functionalization reaction, and the liposomes were mixed or not with 0.75 µM of Osh6(HH/AA) for 10 min. The amount of membrane-bound $_C$Ist2[590–946] and Osh6(HH/AA) was determined using the content of lanes 5 and 6 (100% total), respectively (n = 3–7). Data are represented as means ± SEM with single data points and were analyzed using an Unpaired Mann–Whitney U test (*$P < 0.05$, **$P < 0.01$).

(D) Flotation assay. Liposomes (750 µM lipids) composed of POPC/POPS/$PI(4,5)P_2$/NBD-PE (65:30:5:0.1) were mixed with 0.75 µM of Osh6 or Osh6(HH/AA) in the presence or absence of an equivalent amount of $_C$Ist2[590–946] for 10 min (n = 7). Data are shown as means ± SEM with single data points and were analyzed using an Unpaired Mann–Whitney U test (***$P < 0.001$).

(E) Real-time NBD-PS transfer assays. (i) Osh6 was added to L_{ER} liposomes (200 µM total lipids), containing 10% MPB-PE and 2% NBD-PS, and connected by $_C$Ist2[590–946] (0.5 µM) to L_{PM} liposomes (200 µM), composed of POPC/POPS/$PI(4,5)P_2$/Rhod-PE (63:30:5:2) in the presence of free L_O liposomes (200 µM), composed of POPC/POPS (70:30). A mirror experiment (ii), in which Rhod-PE was incorporated in L_O and not L_{PM} liposomes, was conducted to measure the specific transfer of NBD-PS from L_{ER} to L_O liposomes. These two experiments were also performed without $_C$Ist2[590–946] (iii, iv). The FRET signal has been normalized to determine the amount of NBD-PS (in µM) transferred to L_{PM} or L_O liposomes over time. Some kinetics obtained with Osh6(HH/AA) (Fig. 5B) have also been normalized in the same way to compare the amount of NBD-PS transferred by Osh6 and Osh6(HH/AA) in these assays. Each curve represents the mean ± SEM of several kinetics (n = 3).

(F) NBD-PS transfer rate ($1/t_{1/2}$) measured with Osh6 in conditions (i), (ii), (iii), and (iv). R_{Ist2} corresponds to the Ist2-dependent $\frac{ER\rightarrow PM}{ER\rightarrow O}$ transfer rate ratio

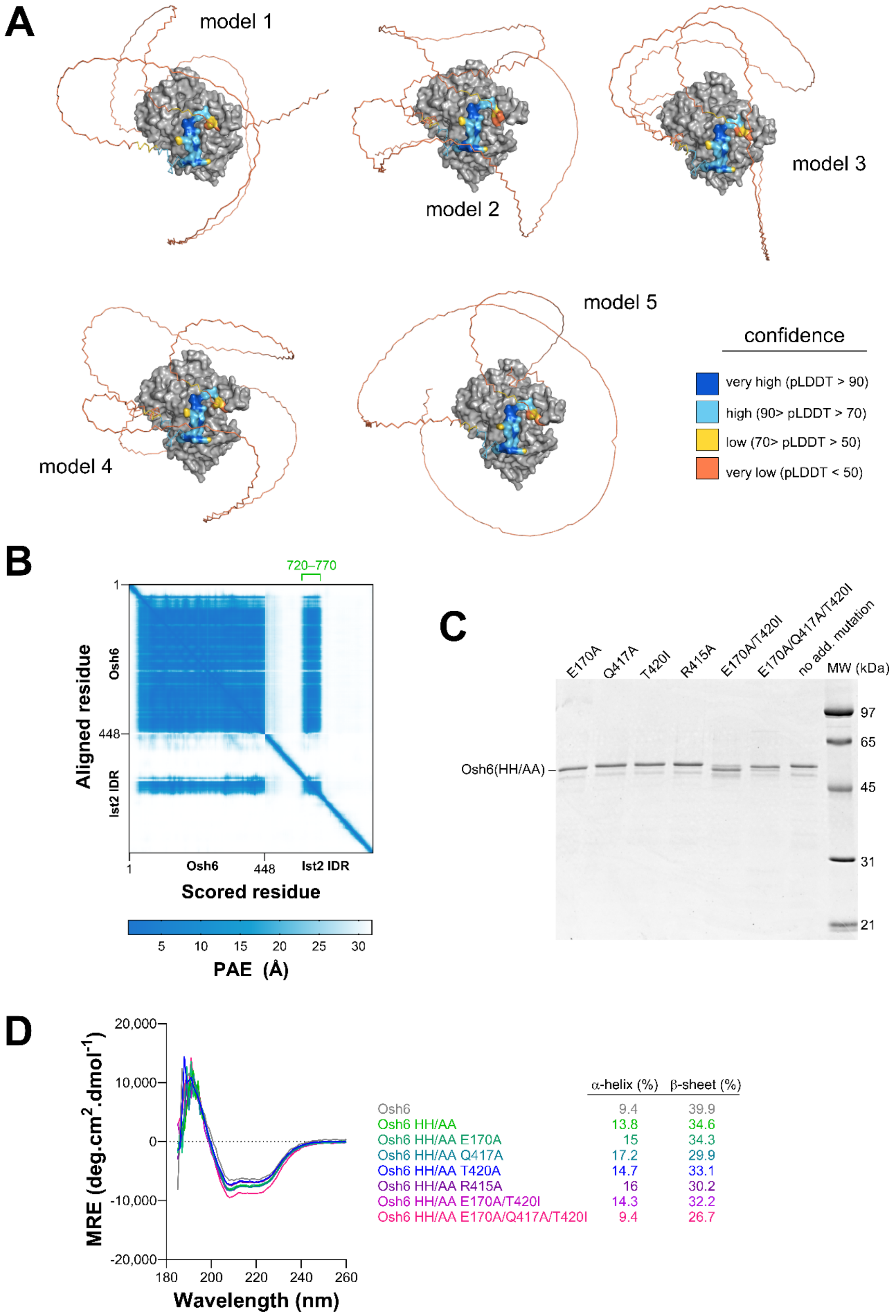
A
model 1
model 2
model 3
model 4
model 5
confidence
very high (pLDDT > 90)
high (90> pLDDT > 70)
low (70> pLDDT > 50)
very low (pLDDT < 50)
B
720–770
Aligned residue
Osh6
Ist2 IDR
1
448
Scored residue
PAE (Å)
5 10 15 20 25 30
C
E170A
Q417A
T420I
R415A
E170A/T420I
E170A/Q417A/T420I
no add. mutation
MW (kDa)
Osh6(HH/AA)
97
65
45
31
21
D
MRE (deg.cm2.dmol-1)
20,000
10,000
0
-10,000
-20,000
180 200 220 240 260
Wavelength (nm)
α-helix (%) β-sheet (%)
Osh6 9.4 39.9
Osh6 HH/AA 13.8 34.6
Osh6 HH/AA E170A 15 34.3
Osh6 HH/AA Q417A 17.2 29.9
Osh6 HH/AA T420A 14.7 33.1
Osh6 HH/AA R415A 16 30.2
Osh6 HH/AA E170A/T420I 14.3 32.2
Osh6 HH/AA E170A/Q417A/T420I 9.4 26.7

Fig. S6. Identification of the Ist2-binding region on Osh6's surface

(A) Five models of the Osh6:Ist2 IDR complex generated by AlphaFold3. Osh6 is represented as a grey surface. For clarity, the N-terminal low complexity sequence of Osh6 (35 residues) has been omitted. The Ist2 IDR is represented in ribbon mode and colored according to the predicted local distance difference test (pLDDT) *per* atom. The Ist2[729–747] segment (minimal Osh6-binding motif) is represented as a surface.

(B) PAE is presented as a two-dimensional plot where the color at coordinates (x, y) represents the predicted position error at residue x if the expected and actual structures were aligned on residue y. The PAE values calculated for residue pairs from Osh6 and Ist2 IDR suggest that the position and orientation of the segment [720–770] of the Ist2 IDR relative to Osh6 are predicted with high confidence (lowest PAE values).

(C) SDS-PAGE analysis of purified Osh6(HH/AA) constructs bearing additional mutations.

(D) Far-UV CD spectrum of purified Osh6(HH/AA) and mutants (6.85–11.88 µM) in 20 mM Tris, pH 7.4, 120 mM NaF buffer at room temperature. The percentages of α-helix and β-sheet, derived from the analysis of each spectrum, are given, as well as the values derived from the crystal structure (PDB ID: 4B2Z) using the DSSP algorithm. MRE, mean residue ellipticity.

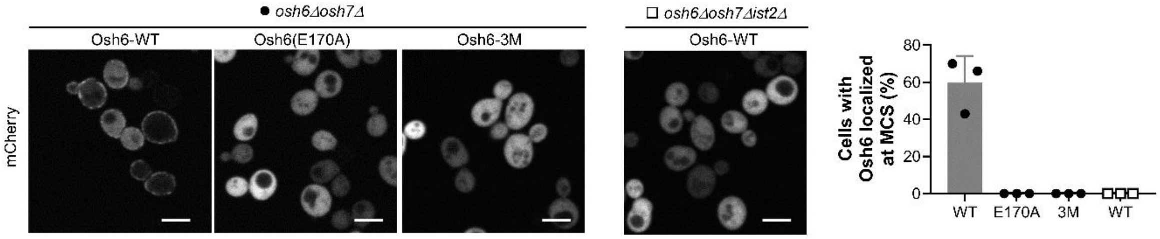

Fig. S7. Osh6 mutants unable to bind to the Ist2 IDR are completely cytosolic in yeast
Localization of Osh6, Osh6(E170A), and Osh6(E170A/Q417A/T420I) (3M), tagged with mCherry, in *osh6Δosh7Δ* cells. Localization of Osh6 in *osh6Δosh7Δist2Δ* cells is shown for comparison. Scale bar, 5 µm. Right plot: percentage of cells with Osh6 at contact sites in *osh6Δosh7Δ* cells (mean of n ≥ 100 cells, analyzed in 3 independent experiments).

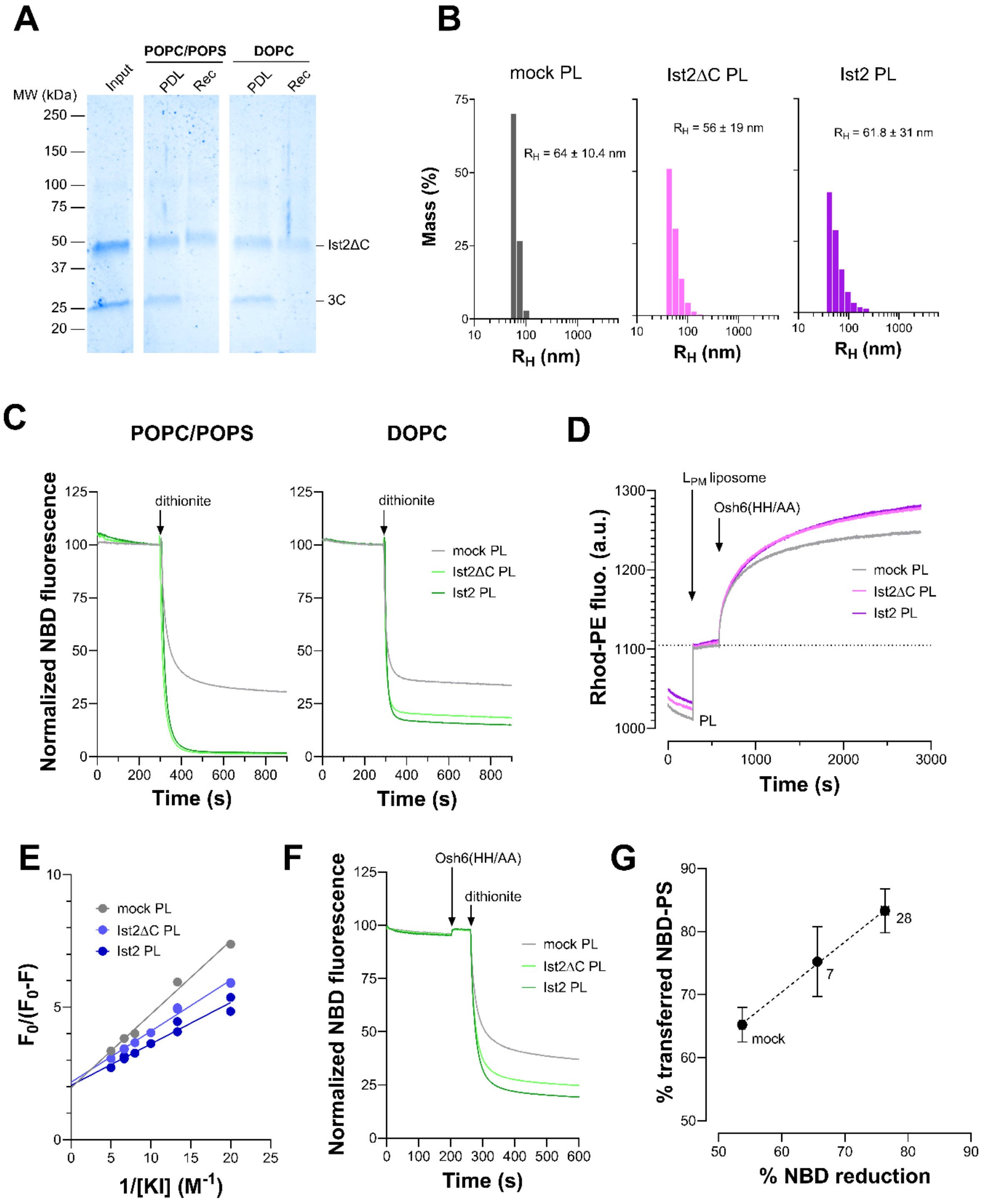

Fig. S8. Osh6-mediated PS transfer is sustained by Ist2 PS scrambling activity

(A) Example of reconstitution. Ist2ΔC was reconstituted in liposomes composed of POPC/POPS/NBD-PS (89:9:2) or DOPC/NBD-PS (98:2) at ~ 1/7000 protein/lipid molar ratio. Therefore, the protein/detergent/lipid (PDL) mixture contains 0.5 µM protein and 3.6 mM lipids. The reconstitution buffer was 50 mM MOPS-Tris, pH 7, and 200 mM NaCl. 'Input' corresponds to the protein sample after affinity purification on streptavidin beads. About 1 µg was loaded on

the gel. PDL, protein/detergent/lipid mixture, before removal of the detergent. Rec, reconstituted samples after detergent removal with Biobeads. Samples were analyzed on a 4–15% TGX precast gel and were stained with Coomassie Blue.

(B) Hydrodynamic radius distribution by mass of mock, Ist2, and Ist2ΔC proteoliposomes diluted in HK buffer at 25 °C.

(C) Scramblase assay. NBD-PS-containing proteoliposomes, in which the Ist2ΔC or Ist2 construct was reconstituted, or protein-free ("mock") proteoliposomes, were treated with dithionite (40 mM) to analyze the movement of NBD-PS between proteoliposome leaflets. Assays were done in 50 mM MOPS-Tris, pH 7, 200 mM NaCl buffer, under stirring at 20 °C. The initial fluorescence was recorded for 300 s before the addition of dithionite to quench NBD-lipids present in the outer leaflet.

(D) Real-time NBD-PS transport assays. These raw kinetics correspond to the normalized curves shown in Fig. 7B.

(E) Collisional quenching of the fluorescence of NBD-PS present in the membrane of mock and Ist2-containing PLs by iodide ions. The data are as modified Stern-Volmer plots (see Materials and Methods). F_0 is the fluorescence intensity of the sample in the absence of quencher, whereas F is the fluorescence intensity at a given iodide ion concentration. The data points were fitted to a linear regression. The y-intercept of the linear regression for "mock" PLs is 1.962 ± 0.206, whereas it is 2.168 ± 0.107 for Ist2 PLs and 2.052 ± 0.157 for Ist2ΔC PLs. This means that ~51 % of NBD-lipids in the membrane of "mock' PLs was accessible to the quencher, whereas ~46% and ~49% of NBD-lipids in the membrane of Ist2-containing proteoliposomes was accessible to the quencher.

(F) Scramblase assay. Osh6 (200 nM) was added to mock, Ist2ΔC, and full-length Ist2 PLs before dithionite treatment (10 mM).

(G) Scramblase and NBD-PS transfer assays were performed as in Fig.7, using proteoliposomes composed of DOPC/NBD-PS (98:2) reconstituted with either no Ist2 (mock PLs) or Ist2 at 7 and 28 $mg.mmol^{-1}$ protein/lipid ratio. The relative percentage of PS transfer determined one hour after the addition of 350 nM Osh6(HH/AA) (mean ± SEM, n = 3–4) is expressed as a function of the percentage of NBD reduction measured 340 s after dithionite addition (mean ± SEM, n = 2–3).

Table S1. Plasmids and inserts used to produce recombinant proteins

Expression plasmids	Construct name	Description	References
pGex2T		Ist2	*(16)*
pGex2T	Ist2[590–946]	Ist2[590–946](S590M) insertion in pGex2T	This work
pGex2T	Ist2[590–936]	Ist2[590–936]](S590M)	This work
pGex2T	Ist2[590–768]	Ist2[590–768](S590C)	This work
pGex2T	Ist2[727–776]	Ist2[727–776]	This work
pGex2T	Ist2[590–946]Δ727-749	Ist2[590–946]Δ727-749(S590C)	This work
pGex2T	$_{C}$Ist2[590–946]	Ist2[590–946](S590C)	This work
pGex2T	$_{C}$Ist2[590–936]	Ist2[590–936](S590C)	This work
pGEX6P-1	ORD^{ORP8}	ORP8[370–807] (XP_054226984.1)	*(12)*
pGEX6P-1	ORD^{ORP8}(HH/AA)	ORP8[370–807] (H514A/H515A)	This work
pDB-ccDB-His-TRX-3C	ORD^{Osh3}	Osh3[605–996]	This work
PGex4T-3	Osh4	Osh4(C98S)	*(72)*
pGex4T-3	Osh6	Osh6	*(8)*
pGex4T-3	Osh6(HH/AA)	Osh6(H157A/H158A)	*(45)*
pGex4T-3	Osh6(H157A/H158A/E170A)	Osh6(H157A/H158A/E170A)	This work
pGex4T-3	Osh6(H157A/H158A/Q417A)	Osh6(H157A/H158A/Q417A)	This work
pGex4T-3	Osh6(H157A/H158A/T420I)	Osh6(H157A/H158A/T420I)	This work
pGex4T-3	Osh6(H157A/H158A/R405A)	Osh6(H157A/H158A/R405A)	This work
pGex4T-3	Osh6 (2M)	Osh6(H157A/H158A/E170A/T420I)	This work
pGex4T-3	Osh6 (3M)	Osh6(H157A/H158A/E170A/Q417A/T420I)	This work
pGex4T-3	Osh6(noC/S190C)	Osh6(C62S/C162S/S190C/C389S)	*(45)*
PGex4T-3	Osh6(noC/T262C)	Osh6(C62S/C162S/T262C/C389S)	*(45)*
pGST3	PHδ1	*R. norvegicus* PLCδ1[1–140](C48S/S61C)	*(44)*
pYeDP60-Ist2-3C-Bad	Ist2	Full-length Ist2	This work
pYeDP60-Ist2ΔC-3C-Bad	Ist2ΔC	Ist2[1–600]	This work

Table S2: Plasmids for yeast experiments used in this study. Note that all plasmids carry ampicillin antibiotic resistance. For mutated versions, the retained codon positions are indicated in brackets.

Plasmids	Alias	Description	References
**pRS416**		*CEN, URA3*	Lab collection
**pOsh6-mCherry**	pAC100	pRS416-based, ADH1pr-Osh6-mCherry	*(16)*
**pCS_39**	pOsh6-mCherry-E170A	ADH1pr-Osh6-mCherry, *E170A*; Mutations inserted by Gibson cloning from a pGex4T-3 plasmid expressing Osh6(E170A) into pAC100 plasmid	This work
**pCS_41**	pOsh6-mCherry-3M	ADH1pr-Osh6-mCherry, *E170A*, *Q417A*, *T420I*; Mutations inserted by Gibson cloning from a pGex4T-3 plasmid expressing Osh6(E170A/Q417A/T420I) into pAC100 plasmid	This work
**pRS413**	pRS413/pEV	*CEN, HIS3*	Lab collection
**pAK75**	GFP-Ist2	pUG34-based *(CEN, HIS3)*, Ist2pr-BFP-Ist2	*(22)*
**pJMD_07**	pIst2	pUG34-based (*CEN, HIS3*), Ist2pr-BFP-Ist2	*(16)*
**pJMD_13**	pIst2[1–877]	Ist2pr-BFP-Ist2[1–877]; Exclusion of [878-946] region from pAK75 recombined into pJMD_07	This work
**pJMD_10**	pIst2[590–946]	Ist2pr-BFP-Ist2[590–946]; Exclusion of [1-590] region from pAK75 recombined into *BamHI*/*XhoI* linearized pJMD_07	This work
**pJMD_30**	pIst2[1–877]PM	Ist2pr-BFP-Ist2[1–877]-CAAX; insertion of CAAX-coding motif by Gibson cloning into pJMD_07	This work
**pCS_45**	pIst2$^{736\text{-}743\Delta}$	Ist2pr-BFP-Ist2 [1–736; 743–946]; Site-directed mutagenesis of pJMD_07	This work
**pJMD_20**	pIst2-short-tail	Ist2pr-BFP-Ist2-[1–590; 705–762; 879–946]; Synthetic gene integrated by Gibson cloning into pJMD_07	This work
**pJMD_33**	pIst2-short-tail+23aa	Ist2pr-BFP-Ist2-[1–590; 682–762; 879–946]; Extension of 23 amino acids [682–704] by Gibson cloning into pJMD_20	This work
**pJMD_34**	pIst2-short-tail+40aa	Ist2pr-BFP-Ist2-[1–590; 665–762; 879–946]; Extension of 40 amino acids [665–704] by Gibson cloning into pJMD_20	This work
**pJMD_36**	pIst2-very-long-tail	Ist2pr-BFP-Ist2-[1–704; 590–704; 705–877; 763-946]; Duplication of [590–704] and [763–877] regions inserted by Gibson cloning into pJMD_20	This work
**pJMD_18**	pIst2-BS-Osh6-ER	Ist2pr-BFP-Ist2-[1–589; 705–762; 590–704; 763–946]; Position change of [705–762] region inserted by Gibson cloning into pJMD_20	This work
**pJMD_19**	pIst2-BS-Osh6-PM	Ist2pr-BFP-Ist2-[1–704; 763–877; 705–762; 878–946]; Position change of [705–762] region inserted by Gibson cloning into pJMD_20	This work
**LactC2-GFP**	pC2lact-GFP	GPDprC2Lact-GFP, *CEN*, *URA3*, Addgene plasmid # 22852	*(80)*

Table S3: Yeast strains used in this study. Numbers after the Δ symbol indicate the starting position or the codon range of the deleted region

***S.cerevisiae* S288 Alias**	**Strains**	**Background**	**Genotype**	**References**
**SGAY1635**	*osh6Δosh7Δ*	SGA Y7039	*MATα can1Δ::STE2pr-LEU2 lyp1Δ ura3Δ0 leu2Δ0 his3Δ1 met15Δ0 osh6Δ::HygMX osh7Δ::NatMX*	*(7)*
**MdPY01**	*ist2Δosh6Δosh7Δ*	SGA Y7039	*MATα can1Δ::STE2pr-LEU2 lyp1Δ ura3Δ0 leu2Δ0 his3Δ1 met15Δ0 ist2Δ::KanMX osh6Δ::HygMX osh7Δ::NatMX*	*(16)*
**VAY2802**	*ist2ΔOSH6-GFP*	BY4742	*MATα his3Δ1 leu2Δ0 lys2Δ0 ura3Δ0 ist2Δ::Hyg OSH6-GFP::KanMX*	*(16)*
**MdPY12**	*ist2Δ OSH6-GFP TCB1-TagRFP*	BY4742	*MATα his3Δ1 leu2Δ0 lys2Δ0 ura3Δ0 ist2Δ::Hyg OSH6-GFP::KanMX TCB1-TagRFP::URA3*	This work
**MdPY08**	*cho1Δ*	BY4742	*MATα his3Δ1 leu2Δ0 lys2Δ0 ura3Δ0 cho1Δ::kanMX*	*(16)*
**MdPY07**	*cho1Δ ist2$^{736–743Δ}$*	BY4742	*MATα his3Δ1 leu2Δ0 lys2Δ0 ura3Δ0 cho1Δ::kanMX ist2Δ736–743*	*(16)*
**MdPY11**	*psd1Δ ist2Δ*	BY4741	*MATa his3Δ1 leu2Δ0 met15Δ0 ura3Δ0 psd1Δ::kanMX ist2Δ::natMX*	*(28)*

Data S1. (separate file)

Excel sheet listing the H-bonds between Osh6's and Ist2's residues identified in the five models of Osh6:Ist2 complex generated by AlphaFold3. A final table reports the number of times each H-bond is identified. The type of H-bonds is named as follows: sm, H-bond between the side-chain of an Osh6's residue and the main chain of an Ist2's residue; ms, m, H-bond between the side-chain of an Ist2's residue and the main chain of an Osh6's residue; ss, H-bond between the side-chain of an Osh6's residue and the side-chain of an Ist2's residue; mm, H-bond between the main chain of an Osh6's residue and the main chain of an Ist2's residue.
